# The relationship between spectral signals and retinal sensitivity in dendrobatid frogs

**DOI:** 10.1371/journal.pone.0312578

**Published:** 2024-11-14

**Authors:** Whitney G. Walkowski, Corinne L. Richards-Zawacki, William C. Gordon, Nicolas G. Bazan, Hamilton E. Farris

**Affiliations:** 1 Neuroscience Center, School of Medicine, LSUHSC, New Orleans, LA, United States of America; 2 Department Cell Biology and Anatomy, School of Medicine, LSUHSC, New Orleans, LA, United States of America; 3 Department of Biological Sciences, University of Pittsburgh, Pittsburgh, PA, United States of America; 4 Department of Ophthalmology, School of Medicine, LSUHSC, New Orleans, LA, United States of America; 5 Department of Otolaryngology & Biocommunication, School of Medicine, LSUHSC, New Orleans, LA, United States of America; University of Akron, UNITED STATES OF AMERICA

## Abstract

Research on visually driven behavior in anurans has often focused on Dendrobatoidea, a clade with extensive variation in skin reflectance, which is perceived to range from cryptic to conspicuous coloration. Because these skin patterns are important in intraspecific and interspecific communication, we hypothesized that the visual spectral sensitivity of dendrobatids should vary with conspecific skin spectrum. We predicted that the physiological response of frog retinas would be tuned to portions of the visible light spectrum that match their body reflectance. Using wavelength-specific electroretinograms (ERGs; from 350-650 nm), spectrometer measurements, and color-calibrated photography of the skin, we compared retinal sensitivity and reflectance of two cryptic species (*Allobates talamancae* and *Silverstoneia flotator*), two intermediate species (*Colostethus panamansis* and *Phyllobates lugubris*), and two conspicuous aposematic species (*Dendrobates tinctorius* and *Oophaga pumilio*). Consistent with the matched filter hypothesis, the retinae of cryptic and intermediate species were sensitive across the spectrum, without evidence of spectral tuning to specific wavelengths, yielding low-threshold broadband sensitivity. In contrast, spectral tuning was found to be different between morphologically distinct populations of *O*. *pumilio*, where frogs exhibited retinal sensitivity better matching their morph’s reflectance. This sensory specialization is particularly interesting given the rapid phenotypic divergence exhibited by this species and their behavioral preference for sympatric skin reflectances. Overall, this study suggests that retinal sensitivity is coevolving with reflective strategy and spectral reflectance in dendrobatids.

## Introduction

Visual systems have evolved in response to a variety of selective pressures, including in the context of natural and sexual selection [1,2]. The result is that across taxa there is extensive variance in several properties of visual processing, as species may exhibit specializations for different levels of photon capture (diurnal vs. nocturnal), spatial resolution, or wavelength sensitivity [2–6]. The latter is the focus of this study. Using a comparative approach with several species of diurnal frogs we tested if retinal physiological responses to stimuli with different wavelengths vary with species’ visual ecology and skin reflectance spectra.

The ancestral diel activity pattern of anuran amphibians is nocturnal, which is still expressed in the overwhelming majority of species [7,8]. This behavior under low light conditions has resulted in elaborate acoustic displays and auditory processing capabilities [9]. However, where diurnality has evolved, it is generally accompanied with the evolution of visual signaling mechanisms, like foot-flagging or conspicuous skin patterns [7]. These visual signals are important in intraspecific communication which function in mate choice behavior, agonistic territorial defense, and parental care [7,10–13]. Thus, it is hypothesized that the visual systems of diurnal frog species should vary with, and potentially be specialized for, these visual signals.

The largest radiation of diurnal frog species belongs to the superfamily Dendrobatoidea, which is made up of two families: Aromobatidae and Dendrobatidae [14,15]. Diurnality is thought to be common to nearly all members of this superfamily [16,17]. Like for other diurnal taxa [18], the increased availability of light facilitates the evolution of both varied visual processing and the production of visual signals across different wavelengths of light, which for humans are perceived as colors [19]. Within Dendrobatoidea, this has enabled rapid phenotypic divergence in skin color patterns resulting in extensive intra- and interspecific variation [20–24]. For a subset of species, this variance in visual patterning manifests as aposematism, or bright conspicuous coloration of the skin which functions as a signal to predators warning of the presence of sequestered toxins [25]. Previous work has shown that the brightness of body coloration, or more accurately termed the intensity of skin reflectance, is positively correlated to toxin defense [21,26]. That is, when compared to those with high toxin concentrations, dendrobatid species that sequester low concentrations typically exhibit a lesser degree of aposematism or lack this signaling mechanism all together [25,27,28]. Therefore, skin reflectance is considered an honest indicator of toxicity within this clade.

The relationship between toxins and skin reflectance suggests that skin reflectance spectra, the wavelengths of light reflected from the skin, should at least be under natural selection from predator visual systems. Just such evidence is found in the aposematic species *Oophaga pumilio*, where the visual spectral sensitivity of potential predators matches the reflectance spectrum from the frogs’ dorsal surface [26]. In contrast to aposematic species, the skin reflectance of non-aposematic species appears to have been selected for crypsis, blending into the visual patterns and wavelengths of their environment to avoid detection by predators [29]. Across these two extremes, species in this clade exhibit a range of strategies for reducing risk from visual predators during diurnal behavior; in this paper we classified frogs as **aposematic** and toxic, **intermediate** with low levels of aposematism and toxins, or **cryptic** and non-toxic. This research explored if these anuran phenotypes could also be associated with physiological differences in frog visual sensitivity?

Previous work has shown that, in addition to natural selection, skin reflectance may also be under the influence of sexual selection [30–33]. Many species of anurans, both nocturnal and diurnal, utilize visual signals during mate choice decisions [34–36]. Given the selective advantage of bright reflectance in aposematic species, mate searching frogs could be expected to prefer conspecifics with conspicuous skin reflectance, reinforcing aposematism while accruing benefits from improved search times [30]. Behavioral data support such preference, where females from spectrally distinct populations of *O*. *pumilio* show associative responses to males with skin spectral reflectance similar to that in females [10,11,13]. Additionally, in another phenotypically diverse, aposematic species, *Dendrobates tinctorius*, there is an observed reduction in acoustic signaling when compared to closely related species; it is hypothesized that this increases the reliance on intraspecific visual signaling [37,38]. Under such circumstances, one outcome could be that the spectral sensitivity of frog visual systems evolves to match that of intraspecific skin reflectance. Thus, in aposematic species we predicted that spectral sensitivity (sensitivity to particular wavelengths of light) to be correlated with species specific body spectral reflectance. In contrast, it is predicted that cryptic species will exhibit less specialized sensitivity to portions of the light spectrum and be more broadly tuned to a wider bandwidth.

To study visual spectral sensitivity, we focus on the retina, the portion of the eye responsible for transducing light stimuli into neurophysiological responses [3]. There are several ways to alter spectral sensitivity in the retina, especially at the level of photoreception, including variations in the molecular structure of the chromophore [39,40], changing the pigment and abundance of oil droplets [41], and altering the amino acid sequence of opsins [42,43]. Analysis of opsins protein sequence and light absorbance spectra, respectively, show these proteins may evolve such that absorbance is correlated to a target’s or signal’s wavelengths [42–44]. Although direct measures of visual pigment wavelength absorbance are quite useful, such assays do not completely explain relative wavelength processing or perception (implied color), which results from physiological responses to transduction and includes subsequent neural circuit computations (e.g., forms of lateral inhibition and/or opponent processing) [45]. Previous studies also show that retinal circuitry downstream from the photoreceptors is involved in spectral signal modulation [46]. This means spectral processing is not limited to the spectrum of visual pigment absorbance alone, requiring assays of subsequent visual process to determine what the retina ‘tells the frog brain’ [47]. Therefore, in this study we used electroretinograms (ERGs) which yield a gross measure of the summed electrophysiological response that light stimuli elicit in the photoreceptors and subsequently the cells within the inner-nuclear layer of the retina (bipolar cells, Müller cells, horizontal cells, and amacrine cells). This method was used to test the hypothesis that retinal spectral sensitivity varies with the spectral reflectance of frog skin. Using a comparative approach, the hypothesis was tested across several species of dendrobatid frogs.

## Materials and methods

### Study species

Prior to using live specimens, species for this study were chosen based on perceived variation in reflectance strategy (aposematic, intermediate, or cryptic) viewed in published photographs of live specimens (Cal Photos, Berkeley). We chose two aposematic species, *D*. *tinctorius* and *O*. *pumilio*, both of which sequester toxins [21,25] and exhibit vast phenotypic variation that has produced distinctive spectral morphologies, “morphs”, throughout their natural geographic ranges [24,48]. For example, *D*. *tincotorius* morphs are distinct between rainforest refugia in the Guyana shield [24]. Similarly, *O*. *pumilio* morph populations differ between islands in the Bocas del Toro archipelago in Panama [48]. Previous work indicates that our intermediate species, *Colostethus panamansis* and *Phyllobates lugubris*, sequester low, perhaps inconsequential, concentrations of alkaloid toxins [25,27]. The cryptic species, *Allobates talamancae* and *Silverstoneia flotator*, are non-toxic [28]. Initial review of photographs of living specimens (Cal Photos, Berkeley) suggested that intermediate species possess morphologically small areas of conspicuous coloration, whereas the cryptic species lack such color signals. In the current study, we used ultraviolet-visible-infrared (UV-Vis-IR) spectroscopy and color calibrated photography to empirically confirm these classifications. Thus, characterization includes measures of wavelength reflectance within and outside of the human visible light spectrum (UV and IR).

### Specimens

Specimens were either reared or wild caught. *O*. *pumilio* and *D*. *tinctorius*, were captive bred in colonies established from wild caught specimens. *Dendrobates tinctorius* specimens were purchased from a commercial vendor (Josh’s Frogs). The ”Patricia” *D*. *tinctorius* morphs used here are an F1 generation from parents imported from the wild in 2017. The “azureus” *D*. *tincotorius* morphs, although once considered to be a separate species (*D*. *azureus*) due to morphologically distinct patterning, are genetically indistinguishable [49]. The *O*. *pumilio* specimens were bred in the Richards-Zawacki Lab at the University of Pittsburgh from specimens collected in the Bocas del Toro archipelago in Panama through the Smithsonian Tropical Research Institute (STRI). Laboratory breeding protocol maintains the morphs of populations from different islands within the archipelago. In this study we used the Bastimentos “Cemetery” and Popas Island “Popa” *O*. *pumilio* morphs. The remaining species were hand captured on Pipeline Road in the Soberania National Park in Gamboa, Panama (*A*. *talamancae*, *C*. *panamansis*, and *S*. *flotator*) or near La Grutta in Bocas del Toro, Panama (*P*. *lugubris*) during a 2021 field season (collection supported through STRI IACUC #SI-200005 and permitted through MiAmbiente # ARB-037-2021). Morphometric data were collected from all specimens and ambient light intensity of the collection site were recorded for field collected specimens (Table 1).

**Table 1:** Morphometric data collected from all specimens used in this study and the ambient light intensities of collection sites for all field collected specimens.

	Aposematic				Intermediate		Cryptic	
	*D*. *t*. (Az.)	*D*. *t*. (Pat.)	*O*. *p*. (Cem.)	*O*. *p*. (Pop.)	*C*. *p*.	*P*. *l*.	*A*. *t*.	*S*. *f*.
**n=** **Female** **Male**	3 3	3 5	7 4	6 5	3 3	3 2	3 4	5 5
**SVL (mm)** **Female** **Male**	33.33 (± 1.7) 29.67 (± 1.2)	25.33 (± 2.0) 21.4 (± 1.7)	18.14 (± .46) 18.58 (± 1.1)	16.5 (± .34) 17 (± .55)	24.22 (±.75) 20.48 (± .68)	19.66 (± 1.6) 19.5 (± 1.5)	22.06 (± .68) 20.22 (± .28)	15.25 (± .50) 14.5 (± .20)
**Weight (g)** **Female** **Male**	2.83 (± .29) 2.47 (± .23)	1.53 (± .38) 0.96 (± .22)	0.57 (± .06) 0.63 (± .06)	0.5 (± .04) 0.42 (± .02)	1.47 (± .11) 0.97 (± .12)	1.56 (± .03) 1.50 (± .02)	1.02 (± .05) 0.76 (± .05)	0.4 (± .07) 0.20 (± .01)
**Light Intensity (Log μmol/m**^**2**^**/sec)** **Female** **Male**	- -	- -	- -	- -	0.18 (± .27) 0.14 (± .28)	0.38 (± .37) 0.69 (± .18)	0.24 (± .26) 0.63 (± .11)	0.86 (± .13) 0.46 (± .11)

### Reflectance analysis

Our overall study design sought to determine differences in the physiological response of the retina to specific wavelengths of light between species that vary in skin spectral reflectance. Again, we chose species based on reflective strategy, aposematic, intermediate, or cryptic, which was initially assessed using open-source photographs (Cal Photos, Berkeley). However, the colors perceived in uncalibrated photographs can vary based on photography environment, like light availability and exposure, or post-processing, such as photo editing and filtering [50]. Therefore, to quantitatively confirm our classifications of reflective strategy for individuals in this study we used two approaches: spectrometry and calibrated photographic analysis of our sample frogs. Spectrometer measurements are useful because they provide precise measurements of wavelength reflectance. However, they do not consider the physical area occupied by each reflectance measurement. Therefore, we included calibrated photography to assess overall reflectance scaled for morphological area.

### Spectrometer

We used a BLK-C spectrometer outfitted with a SL1-SL3 Combo lamp setup to measure the spectral reflectance of frog surfaces (StellarNet, Inc.), particularly the dorsal, lateral, axillary, and inguinal regions of each frog. Any distinct morphological markings (e.g., a prominent stripe or spot) were also measured within each region. All spectral reflectances were calculated as a percent of reflectance compared to the RS50 (StellarNet, Inc.) white standard. Curves were captured (SpectraWiz;StellarNet, Inc.), saved (TRM files) and imported to Spectragryph (Dr. Friedrich Menges Software-Entwicklung; Spectroscopy Ninja) for analysis of the peak reflectance (nm), full width half maximum (FWHM; nm), area under the curve from baseline (AUC; % * nm), and average reflectance (%) across the entire spectrum (S1 Fig). Peak was defined as the wavelength (nm) with maximum reflectance. FWHM was calculated as the distance between the wavelengths (nm, bandwidth) on either side of the peak where half of the maximum reflectance occurred. The AUC measurement was the integral of the reflectance curve. The average reflectance (%) is the mean of all reflectance measurements across the entire spectrum.

### Calibrated photography

Animals were photographed using a Canon Rebel T2i SLR camera fitted with a 105mm macro lens (Sigma). A Calibrite ColorChecker Passport (x-rite and Pantone) was included in each photo, which served as a scale bar and color calibration tool. To standardize the color in all photos, we used the ColorChecker DNG profile manager software to automatically detect the ColorChecker Passport and generate color profiles, which were then applied to the RAW photo files in Adobe Lightroom. We then used Adobe photoshop to measure the total dorsal and lateral surface areas of each frog. The dorsal surface was defined rostrally by the snout, caudally by the vent, and laterally by the dorsolateral margin. The lateral surface was defined rostrally by the snout, caudally by the inguinal region, superiorly by the dorsolateral margin, and inferiorly by the labial margin. The area occupied by morphological markings, distinguished visually as distinctive color regions, was measured then calculated as a percentage of the total surface area (area of marking [mm^2^] / total area of dorsal or lateral surface [mm^2^]). We measured the RGB color code for each marking, then used its sum to calculate the total reflectance of that area (R+G+B) [51]. To calculate the scaled reflectance of each frog we multiplied the total reflectance for each marking by the area it occupied, then summed the scaled reflectances of the dorsal and lateral surfaces.

### Electroretinograms (ERG)

All ERGs were conducted under photopic conditions with a background luminance of 0.5μmol/m^2^/sec produced by Absolute Series Lighting (Waveform Lighting), a light source which spans the UV-Vis-IR spectrum and approximates the natural sunlight spectrum. The recording arena was a ‘light-tight’ sound booth (Industrial Acoustic Company, Inc.) surrounded by a Faraday cage. For each experiment, a frog was paralyzed using an intramuscular injection of succinylcholine chloride (15 μg/g; Sigma–Aldrich) and then placed on a wet paper towel to allow for cutaneous respiration. We applied 1% Tropicamide Ophthalmic Solution (Akorn, Inc.) to the frog’s cornea to dilate the pupil. Stainless steel subdermal needle electrodes provided grounding and indifferent recordings. ERG responses were recorded using a silver chloride corneal electrode. The responses were amplified (GRASS P511), filtered (1–100 Hz), and digitized (Cambridge Electronic Design 1401) for offline analysis.

Stimuli were generated by a Xenon light source (Oriel Instruments) and delivered via fiber optics to the cornea of one eye. A Uniblitz shutter (Model VMM-D1) gated the 5 ms-duration flashes. Neutral density filters (Melles Griot) controlled light intensity. Experiments began with four ‘no light’ flashes enabling correction for any DC shift in the recordings. The response at each light intensity consisted of the average of four flashes. Note that on occasion averages included only three traces because of spurious noise during response to one flash. We allowed the retina to recover to baseline for 30-60sec between flashes at the same intensity and wavelength, where longer intervals were used for higher intensity stimuli. When moving to a new wavelength or intensity step we used a 60-120sec interval, again using longer intervals for more high intensity light. This established protocol was used to prevent any retinal adaptation [6,52,53]. At each intensity step light intensity was increased by removing neutral density filters. Within each intensity step, wavelength-specific pass interference filters (Melles Griot or Edmunds Optics) produced stimuli at 400nm, 450nm, 500nm, 550nm, 600nm, or 650nm (± 10nm for each filter). We randomized the order of wavelength stimuli presentation within and between experiments. For a subset of experiments in the aposematic species (*D*. *tinctorius* and *O*. *pumilio*) a UV pass interference filter (Edmunds Optics) was used to deliver a 350nm ± 10nm light stimulus. Light stimuli and background luminance were calibrated using a LI-COR light meter (Model LI-189 with photometric probe; Lincoln, NE). All methods were approved by LSUHSC’s IACUC (#3667).

### ERG analysis

For each intensity step and wavelength the amplitude (μV) of the ERG b-wave (likely indicative of bipolar cell responses [54,55]), was measured. The b-wave responses were chosen (rather than the a-wave) due to their relatively higher signal to noise ratio in the recording, allowing for more accurate determination of smaller amplitude responses around threshold (S2 Fig). B-wave amplitude at each wavelength was normalized to the maximum b-wave amplitude for each animal (b-wave amplitude/maximum b-wave amplitude). This allows us to control for variation between the amplitude of the recordings, as the 10% threshold for each individual would not shift with the amplitude of the response but instead with the maximum response for that individual. These data were plotted as relative amplitude as a function of log light intensity (V-Log[I]) and analyzed a least-squares fit of the standard Boltzmann function [56]:

$$\mathrm{Relative}\;b-\mathrm{wave}\;\mathrm{amplitude}=\frac{A_{1}-A_{2}}{1+e^{\frac{(\mathrm{flash}-{\mathrm{flash}}_{2})}{\tau }}}+A_{2}$$

Here, the starting amplitude is “A_1_” and equal to 0 and the ending amplitude is “A_2_” and equal to 1. The variable “flash” is equal to the log light intensity of each intensity step and the “flash_0_” is equal to the light intensity that yields 50% of the maximum b-wave response. Lastly, “τ” is the slope of the function. Interpolating from the fit, threshold was defined as the light intensity that elicited b-wave amplitude at 10% of the maximum response for each animal at each wavelength [55].

### Statistical analysis

Spectral Reflectance Data: Principal component analysis (PCA) assessed the relatedness of species (and morphs) spectral reflectance curves (curve metrics: peak, FWHM, AUC, and average reflectance) for all the morphological regions and markings sampled. Analysis was conducted using R packages FactoMineR, factoextra, and corrplot [57–59]. A PCA graph was generated depicting the two components (PC1 and PC2) that accounted for the greatest amount of variation in the dataset.

For parametric comparisons, all data met the statistical assumptions of homogenous variance and normality as assessed by a Lavene’s test and Kolmogorov-Smirnov, respectively. We used a one-way ANOVA to analyze the differences in photographic scaled reflectance between species. For ERG responses, two-way ANOVAs were used to test for significant differences in retinal threshold for the presented wavelengths when compared across sexes, species, reflective strategies, and morphs. The alpha level was set at 0.05 for all ANOVAs. For any post-hoc pairwise comparisons we used a Bonferroni correction for multiple comparisons. Statistical analysis and figure generation was completed in RStudio using the ggplot2 and plyr packages [60,61].

## Results

### Reflectance

**Fig 1:**
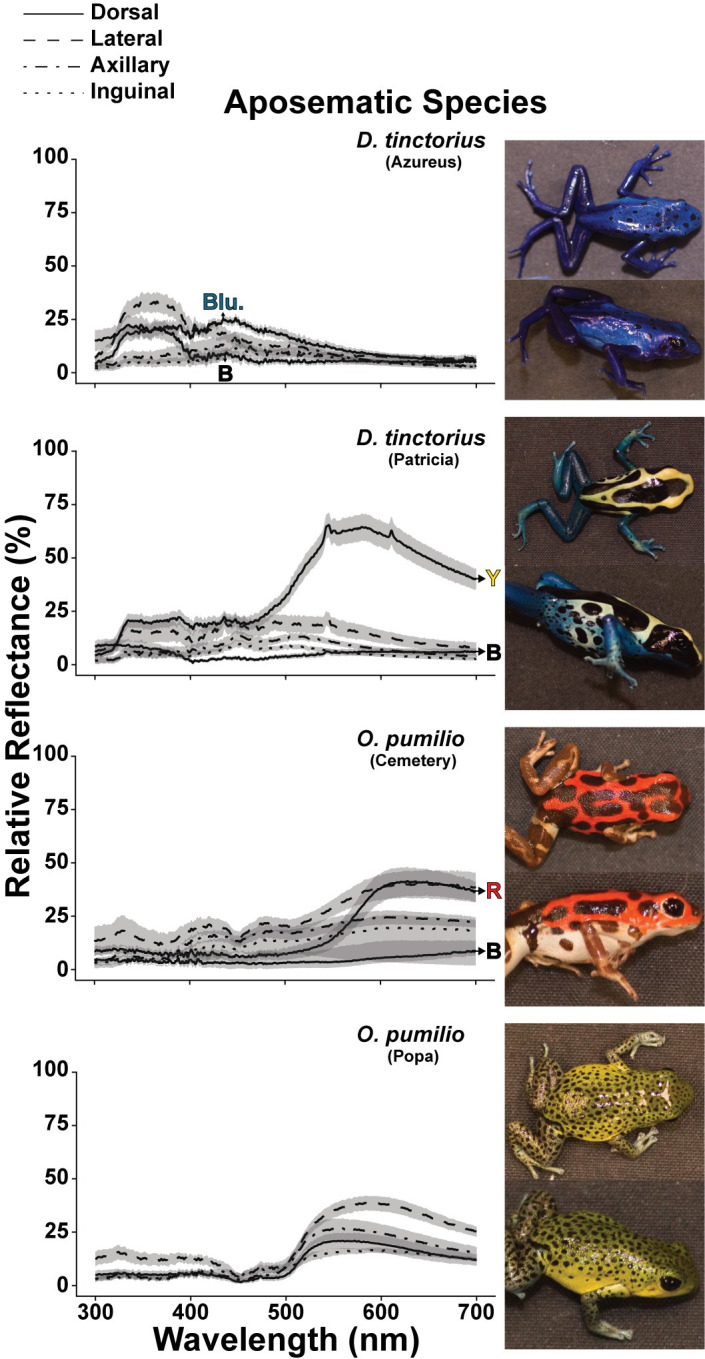
Reflectance data for morphs within aposematic species, *D*. *tinctorius* and *O*. *pumilio*. Lines indicate the average relative reflectance (%) across the wavelength (nm) spectrum. Shading around each line indicates the S.E. of each dataset. Morphological regions are differentiated by line type, where solid lines are dorsal, dashed are lateral, dash-dot are axillary, and dotted are inguinal measurements. Arrows point from a subset of reflectance curves to filled letters, which correspond to subregions of distinct colors, as detected by the human eye (Blu.= blue, B= black, Y= yellow, and R=red). For all quantitative analysis of spectrometer data refer to S1 Table. Example, dorsal and lateral photographs for morphs are included to the right of each reflectance graph. Quantitative analysis of photographs which scales reflectance measurements by morphological region can be found in Fig 4.

**Fig 2:**
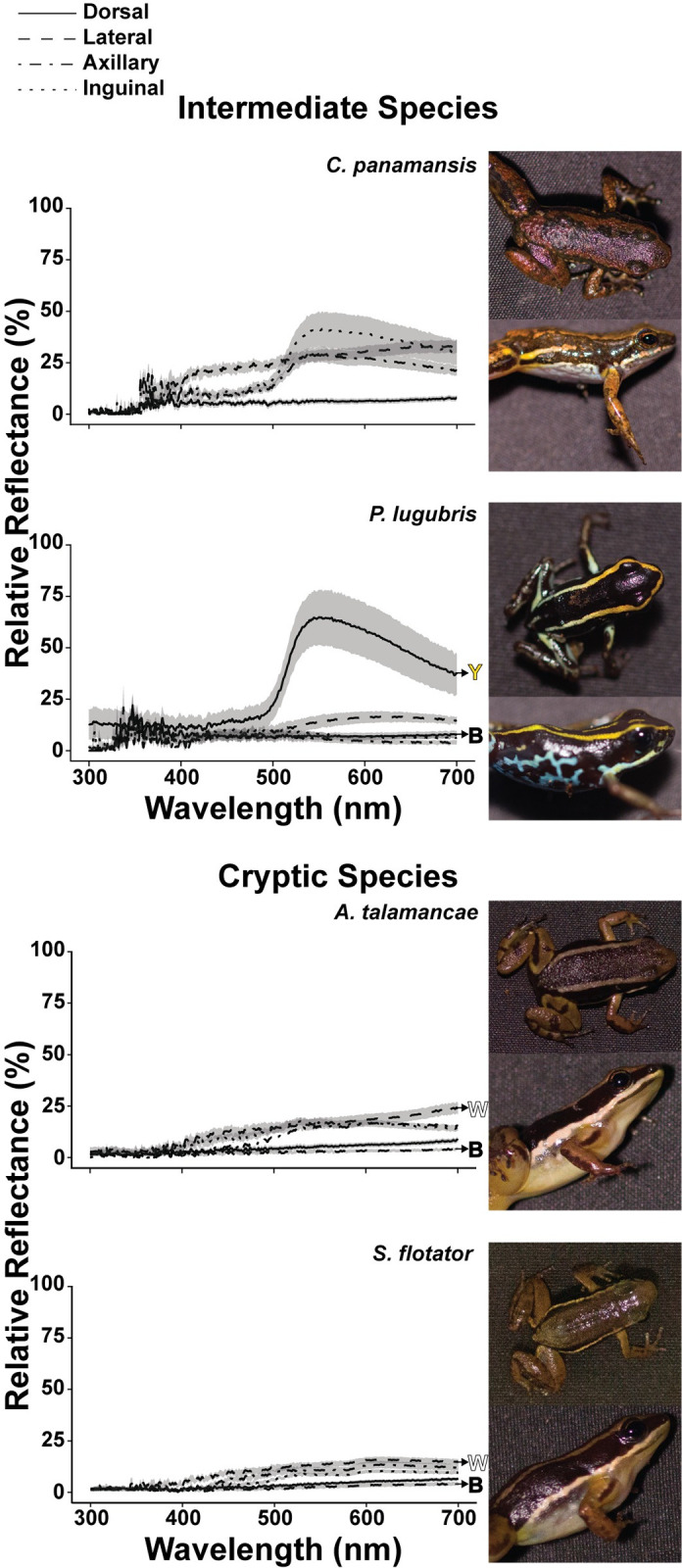
**Reflectance data for intermediate species, *C*. *panamansis* and *P*. *lugubris*, and cryptic species, *A*. *talamancae* and *S*. *flotator*.** Lines indicate the average relative reflectance (%) across the wavelength (nm) spectrum. Shading around each line indicates the S.E. of each dataset. Morphological regions are differentiated by line type, where solid lines are dorsal, dashed are lateral, dash-dot are axillary, and dotted are inguinal measurements. Arrows point from a subset of reflectance curves to filled letters, which correspond to subregions of distinct colors, as detected by the human eye, (Y= yellow, B= black, and W= white). For all quantitative analysis of spectrometer data refer to S1 Table. Example, dorsal and lateral photographs for species are included to the right of each reflectance graph. Quantitative analysis of photographs which scales reflectance measurements by morphological region can be found in Fig 4.

Spectrometry and color calibrated photography confirmed that the individuals from the subject species used here could indeed be classified into one of the three reflectance strategies: aposematic, intermediate, or cryptic. In initial qualitative examination, the spectral reflectance curves of aposematic and intermediate species, collected via spectrometer, appeared distinct from the reflectance curves of the cryptic species (Figs 1 and 2). Aposematic and intermediate species reflectance curves showed obvious peaks, while cryptic species had relatively flat reflectances (Figs 1 and 2; also see AUC and average reflectance data in S1 Table). We also found that aposematic *D*. *tinctorius* and intermediate *C*. *panamansis* and *P*. *lugubris* possess morphological regions that exhibited peak reflectances in the UV spectrum (< 400nm; see peak reflectance data in S1 Table). Cryptic species and the aposematic *O*. *pumilio* lacked peak reflectances at UV wavelengths.

**Fig 3:**
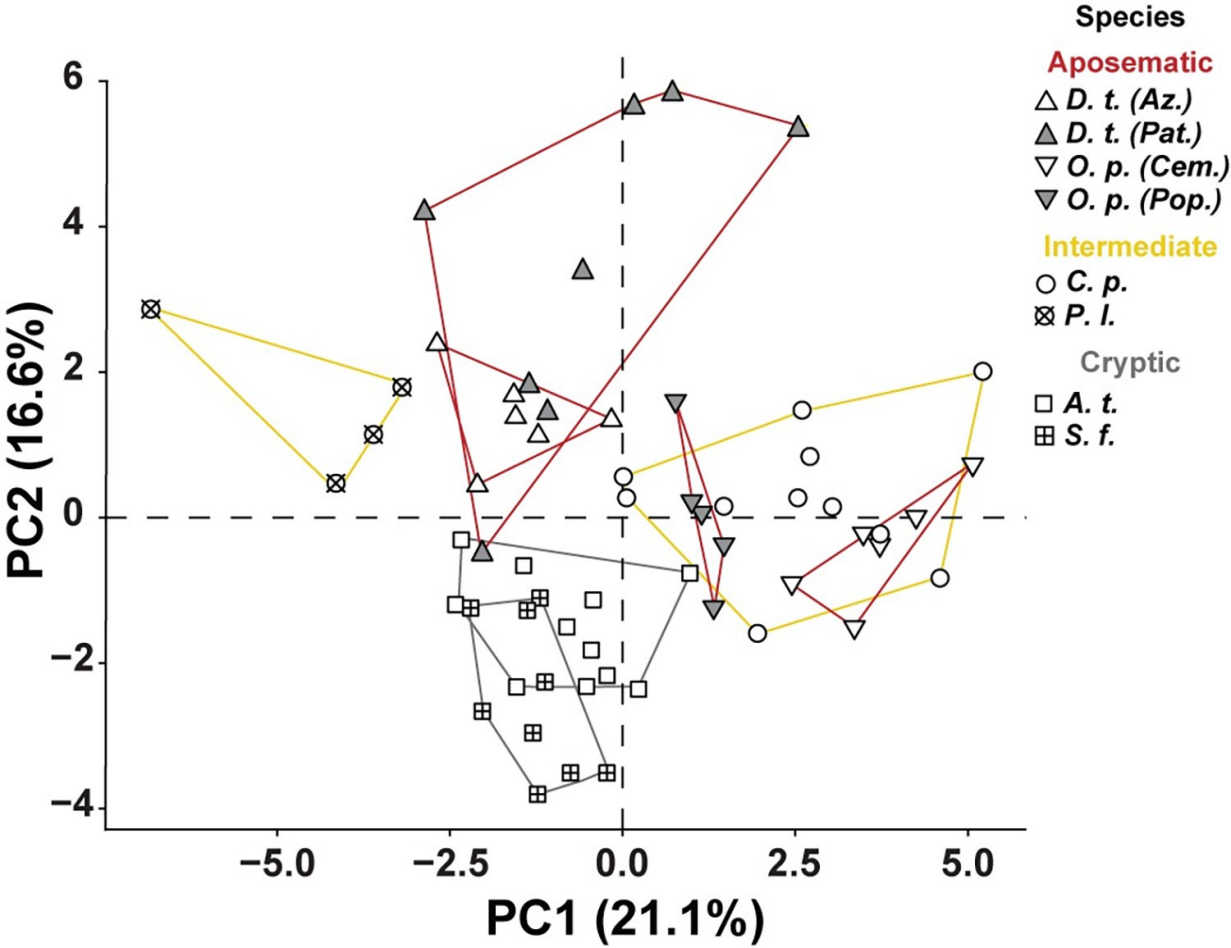
A principal component analysis of spectral data (peak reflectance, FWHM, AUC, and average reflectance) collected from dendrobatid species and/or morphs. *D*. *tinctorius* “Azureus” (*D*. *t*. Az.), *D*. *tinctorius* “Patricia” (*D*. *t*. Pat.), *O*. *pumilio* “Cemetery” (*O*. *p*. Cem.), *O*. *pumilio* “Popa” (*O*. *p*. Pop.) *C*. *panamansis* (*C*. *p*.), P. lugubris (*P*. *l*.), *A*. *talamancae* (*A*. *t*.), *S*. *flotator* (*S*. *f*.). We found PC1 accounted for 21.1% of variation in the dataset, while PC2 accounted for 16.6%. Lines demark the distribution of each species and/or morph. Line color is associated with our hypothesized reflective strategies (red = aposematic, yellow = intermediate, grey = cryptic).

Analysis of spectral reflectance data collected across all morphological regions was accomplished using principal component analysis to test if a specimen’s reflectance curve sorted species by their hypothesized reflectance strategies (Fig 3). When PC1 (21.2% of the variance in dataset) and PC2 (16.6% of the variance in dataset) were plotted, we found that the PCA distribution of the cryptic species’ reflectance data showed extensive overlap with each other and almost no overlap with any other, suggesting these two species reflective strategies are most like one another and different from aposematic and intermediate strategies. For the intermediate species, whose strategy includes low levels of aposematism, some overlap with other species in the PCA analysis might be expected. This is, indeed, what was found: one species grouped with an aposematic species (See *C*.*p*. in Fig 3), while the other exhibited a distribution unique from cryptic and aposematic species (See *P*.*l*. in Fig 3). Regarding aposematic morphs, the distributions of the two aposematic *D*. *tinctorius* morphs were largely distinct from the other species, but overlapped completely with each other, despite appearing visually distinct from one another (See photographs in Fig 1). In contrast, there was no overlap between aposematic *O*. *pumilio* morphs. Yet, as mentioned above, both morphs grouped within the distribution of the intermediate *C*. *panamansis*. Overall, this pattern of distinct cryptic reflectance data and a partial overlap of aposematic and intermediate reflectance data support our categorization of cryptic species.

**Fig 4:**
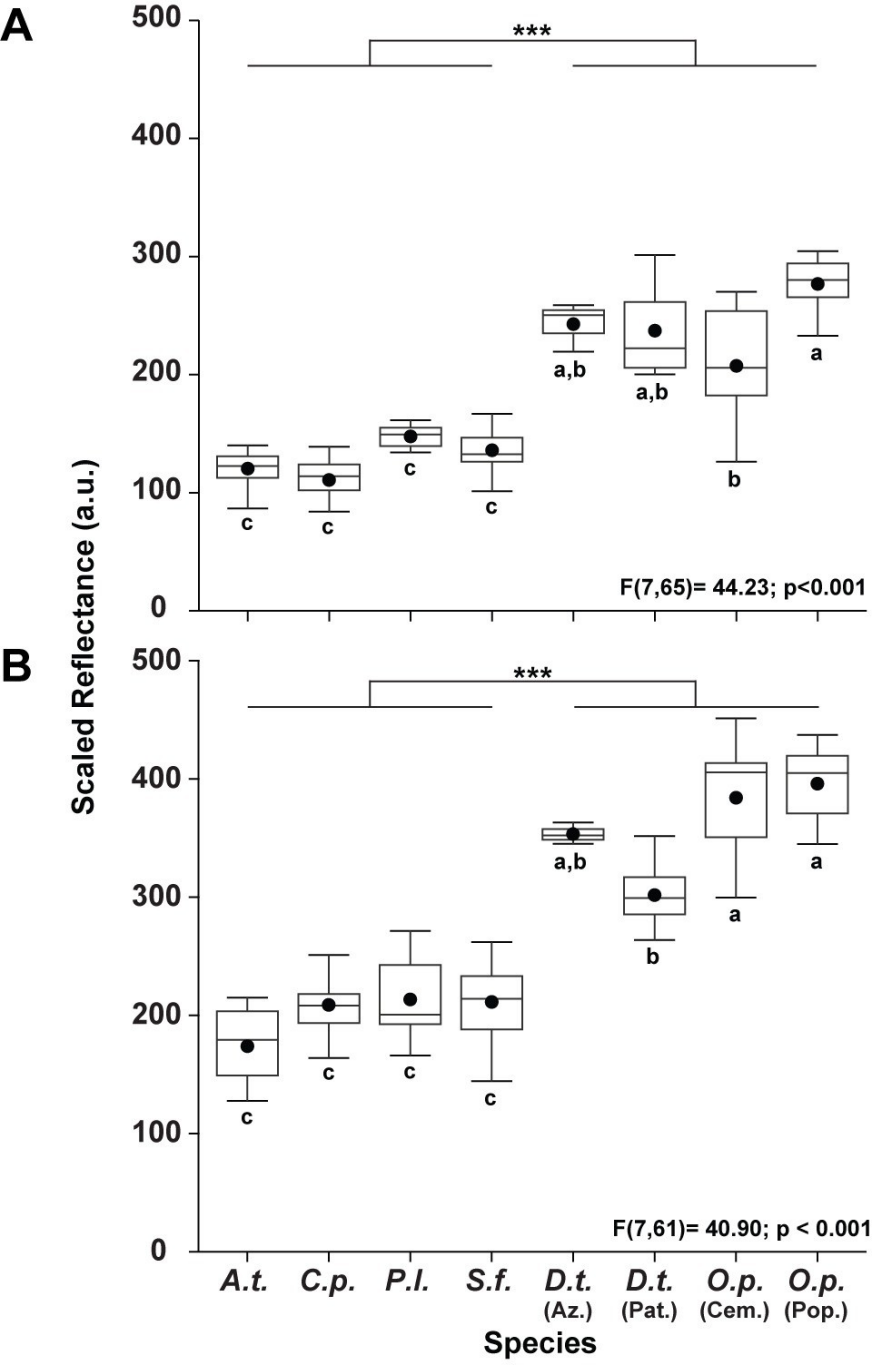
A boxplot illustrating the scaled reflectance of the dorsal (A) and lateral (B) surfaces of the frog species used in this study. *A*. *talamancae* (*A*.*t*.), *C*. *panamansis* (*C*.*p*.), *P*. *lugubris* (*P*.*l*.), *S*. *flotator* (*S*.*f*.), *D*. *tinctorius* azureus morph (*D*.*t*. [Az.]), *D*. *tinctorius* Patricia morph (*D*.*t*. [Pat.]), *O*. *pumilio* Cemetery morph (*O*.*p*. [Cem.]), and *O*. *pumilio* Popa morph (*O*.*p*. [Pop.]). The values displayed in arbitrary units (a.u.) represent a summation of total reflectance (R+G+B) scaled for body surface area. The circles indicate the data means. Lines within the boxes are the medians. Error bars illustrate the range of the dataset. Box size is indicative of the lower (25^th^) and upper (75^th^) quartiles. Within each graph, species that share the same lowercase letters are not significantly different from one another.

Although spectrometer data yielded high precision measurements of reflectance for single points on the frog’s skin, they did not allow for consideration of the morphological area occupied by each reflectance measurement. Therefore, to further assess if our hypothesized reflectance strategies were supported by overall total reflectance, we analyzed color calibrated photographs of frogs. Results from photograph analysis indicate a significant main effect of species on scaled body reflectance for both dorsal (F [7,65] = 44.23; p < 0.001) and lateral (F [7,61] = 40.90; p < 0.001) regions (Fig 4A and 4B). A Tukey’s HSD post-hoc analysis indicated that species categorized as cryptic, *A*. *talamancae* (*A*. *t*.) and *S*. *flotator* (*S*. *f*.), and intermediate, *C*. *panamansis* (*C*. *p*.) and *P*. *lugubris* (*P*. *l*.), had significantly lower scaled reflectances for both dorsal and lateral regions when compared to the morphs of aposematic species, *D*. *tinctorius* (*D*. *t*.) and *O*. *pumilio* (*O*. *p*.) (Fig 4A and 4B). We also found significant differences between aposematic morphs, in which *O*. *pumilio* Cemetery morphs, although more reflective than cryptic and intermediate species, had significantly lower dorsal reflectances than the other aposematic morphs (Fig 4A). Similarly, *D*. *tinctorius* Patricia morphs exhibited lower overall lateral reflectances when compared to the other aposematic species (Fig 4B), but again were more reflective than both cryptic and intermediate species. Taken together, cryptic species, which lack toxins [28], had similar reflectance curves to one another and were less reflective than aposematic species (Table 2). Conversely, toxic aposematic species [25] had distinct reflectance curves from cryptic species and exhibited a greater degree of overall reflectance (Table 2). Lastly, intermediate species, which sequester low levels of toxins [25,27], possessed reflectance curves that are distinct from cryptic species but are similar in terms of total reflectance (Table 2). Thus, these independent approaches to evaluating skin reflectance supported the reflectance strategy categories used below to assess functionally related variance in retinal responses.

**Table 2:** A data summary of toxicity, spectral analysis, total reflectance, and UV reflectance in dendrobatid species. The bolded line indicates grouping of similar data.

	Aposematic		Intermediate		Cryptic	
	*D*. *t*.	*O*. *p*.	*C*. *p*.	*P*. *l*.	*A*. *t*.	*S*. *f*.
**Toxic** **[25,27,28]**	Yes	Yes	Yes (low)	Yes (low)	No	No
**Spectral** **Analysis**	Distinct	Overlap with *C*. *p*.	Overlap with O. *p*.	Distinct	Overlap with A. *t*.	Overlap with S. *f*.
**UV** **Reflectance**	Yes	No*	Yes	Yes	No	No
**Total Reflectance**	High	High	Low	Low	Low	Low

*O. *pumilio* does not possess UV reflectance and is therefore dissimilar from surrounding species

### Threshold comparisons

**Fig 5:**
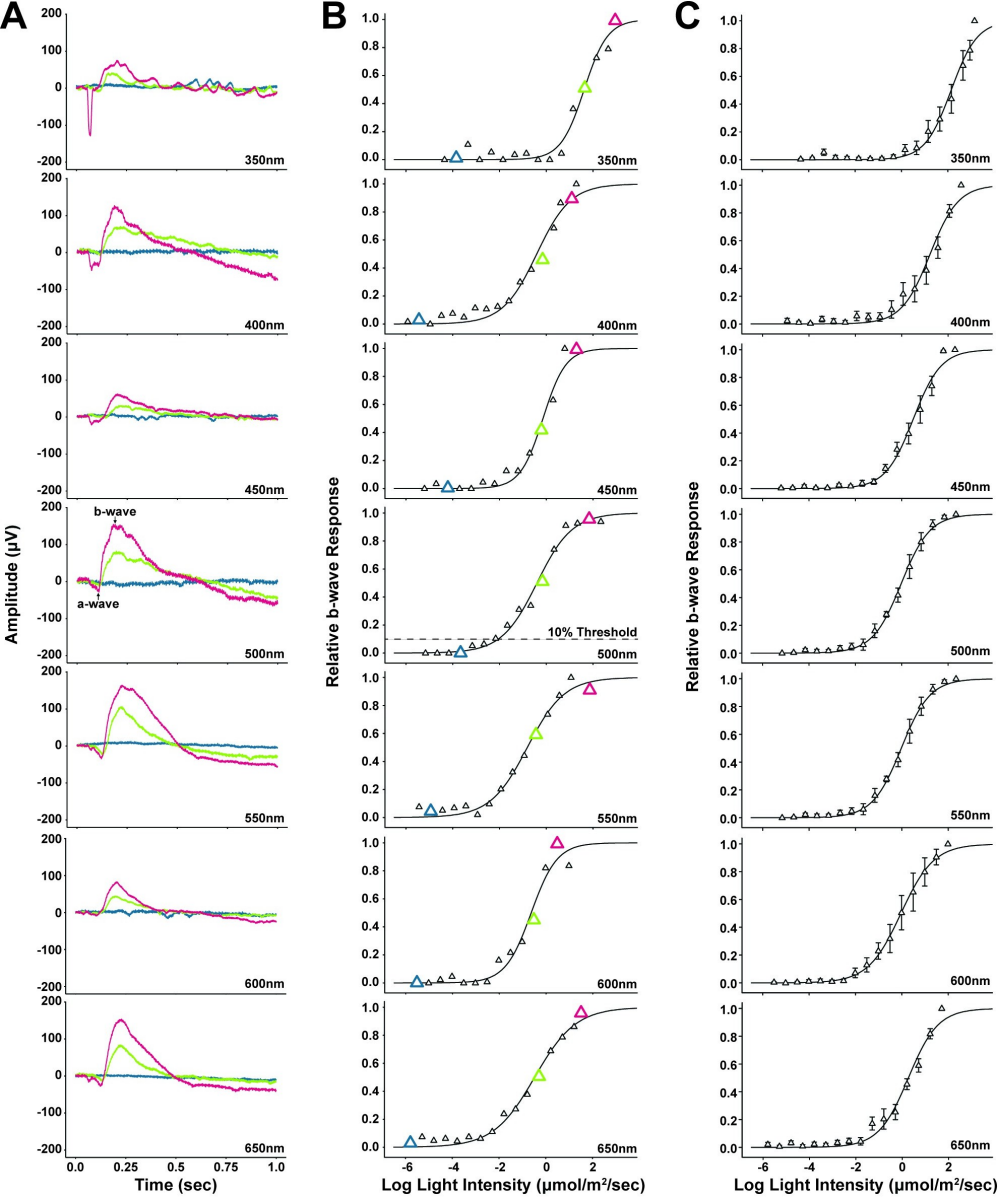
**ERG data for *D*. *tinctorius* (Az.) (A)** Each graph shows example raw ERG traces from a single individual at a specific wavelength (nm) which is labeled in the lower-right corner of each graph. The three traces within each graph show ERG responses to pre-threshold (blue), mid-curve (yellow), and near-saturation (red) light intensities. The arrows on the 500nm graph show typical a-wave and b-wave responses; in other graphs, these responses are unlabeled but can be seen. **(B)** Each graph displays the V-log(I) curves generated from relative b-wave responses (triangles) across light intensities for the same individual shown in figure A_1_. Again, wavelengths (nm) are labeled in the lower-right corner. The outlined, enlarged triangles correspond to the pre-threshold (blue), mid-curve (yellow), and near-saturation (red) responses displayed in Figure A_1_. On the 500nm graph, the light intensity (μmol/m^2^/sec) at which the dotted and solid lines meet is considered the 10% threshold response; this response is unlabeled in other graphs but was calculated for all wavelengths. **(C)** Each graph displays the V-log(I) curves generated from mean (± S.E.) relative b-wave responses (triangles) across light intensities for all *D*. *tinctorius* (Az.) specimens. The wavelengths (nm) are labeled in the lower-right corner of each graph.

ERG responses were reliably generated across the spectrum for all species tested, generating a typical ERG waveform [6], with the amplitude of the b-wave increasing with light intensity (Fig 5A and 5B; Also see S3–S9 Figs). Relative b-wave amplitude (b-wave amplitude [*μ*V] / maximum b-wave amplitude [*μ*V]) as a function of light intensity for each ERG experiment generated a V-log(I) curve that could be fit with a Boltzmann function (Fig 5B; S3–S9 Figs), allowing calculation of threshold for each individual frog at each wavelength (Fig 5B, see 500nm graph). While threshold responses were collected from individual frogs, average relative b-wave responses (± S.E.) illustrated the low amount of variation in the datasets (Fig 5C; S3–S9 Figs), enabling comparison of thresholds across wavelengths (e.g., spectral tuning).

**Fig 6:**
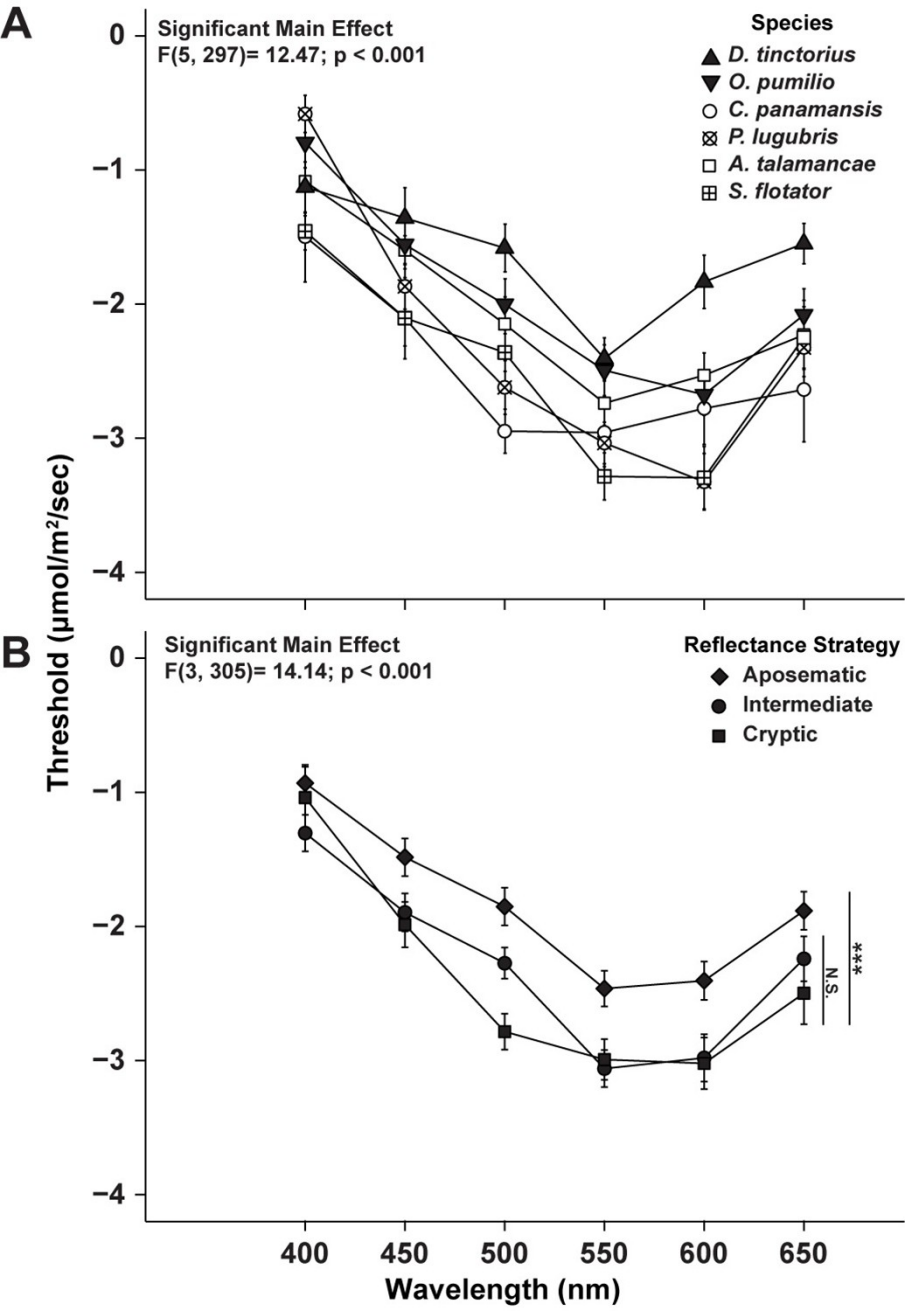
**(A) Relationship between retinal threshold response and wavelength across species.** Points indicate the mean threshold response (± S.E.). A statistically significant main effect of species was found. Filled symbols indicate the combined threshold responses of species that have distinct morphs. Pairwise comparison results indicating statistically different species can be found in S2 Table. No significant interaction between wavelength and species was detected (p = 0.49). **(B) Relationship between retinal threshold response and wavelength across reflectance strategy.** Points indicate the mean threshold response (± S.E.). A statistically significant main effect of reflectance strategy was found. Vertical lines and asterisks denote that cryptic and intermediate species have statistically lower retinal thresholds across the spectra when compared to aposematic species but are not significantly different from one another. No significant interaction between wavelength and reflectance strategy was detected (p = 0.95).

Comparison of tuning curves of all species yielded significant main effect of species (F[5,297] = 14.49; p < 0.001), but no significant interaction between wavelength and species (Fig 6A), as all species were most sensitive to 550-600 nm range with similar elevations in threshold above and below this range. A pair-wise comparison showed that *D*. *tinctorius* had higher retinal threshold responses than all the other species sampled and *O*. *pumilio* exhibited higher thresholds than both *C*. *panamansis* and *S*. *flotator* (S2 Table). It should be noted that the distinct morphs of aposematic species were combined for this analysis, as our initial expectation was that that interspecific variation would be greater than intraspecific. However, *O*. *pumilio* showed significant intraspecific variation (discussed below), because of this these significant findings could be conservatively skewed. Therefore, we included an additional analysis with the *O*. *pumilio* and *D*. *tinctorious* morphs considered individually. This showed a significant effect of species but no interaction (F[7,271]= 12.72; p<0.001; S10 Fig). Pairwise comparisons from these analyses can be found in our supplemental material (S3 Table). The final significant finding for species level analysis was that intermediate species *C*. *panamansis* had significantly lower threshold responses when compared to cryptic species *A*. *talamancae* (Table 2). When species level analysis is taken as a whole, we found that there are differences in overall light sensitivity (across the whole spectrum) between dendrobatid species, but that these differences did not indicate varied tuning to specific wavelengths. We found no effect of sex for any of the species examined (S4 Table).

The species level analysis, however, suggested differences between the retinal response of animals that exhibited different reflective strategies: cryptic, intermediate, and aposematic species. There was a main effect of reflective strategy (F[3,305] = 14.14; p <0.001; Fig 6B) without a significant interaction with wavelength. Pairwise comparison showed that aposematic species had overall higher retinal thresholds than both cryptic and intermediate species, which did not differ from one another. This means that the retinas of aposematic species required higher intensities of light to reach the 10% threshold response than their cryptic or intermediate counterparts. These differences in threshold responses still did not indicate wavelength specific differences, but instead an overall increase or decrease in retinal sensitivity across the spectrum.

**Fig 7:**
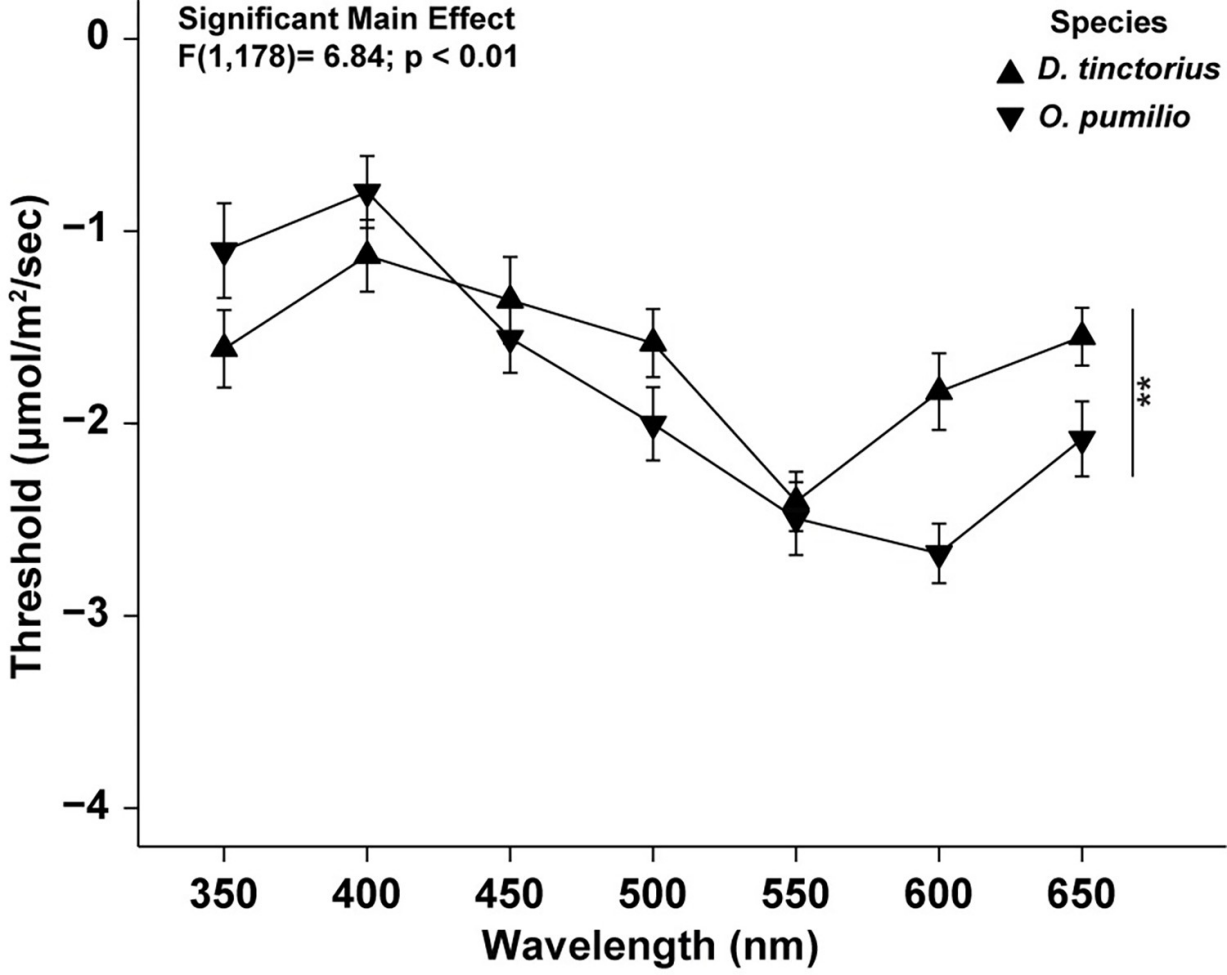
Relationship between retinal threshold response and wavelength across species. Points indicate the mean threshold response (± S.E.). Each species’ average threshold responses are from two distinct morphs. A statistically significant main effect of species was found. Vertical lines and asterisks denote that *O*. *pumilio* have statistically lower retinal thresholds than *D*. *tinctorius* across the spectra. No significant interaction between wavelength and species was detected (p = 0.10).

**Fig 8:**
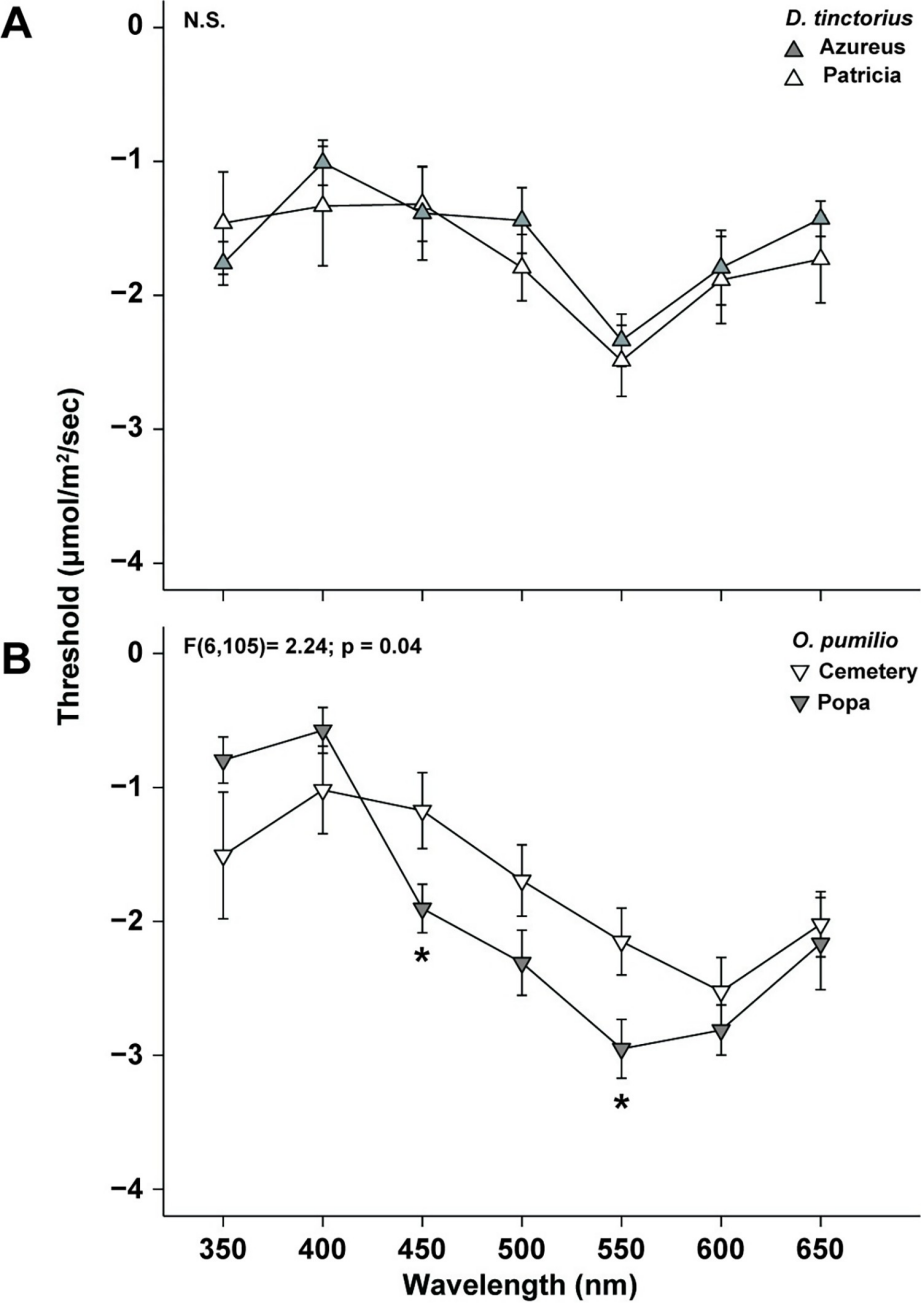
**(A) Relationship between retinal threshold response and wavelength across *D*. *tinctorius* morphs.** Points indicate the mean threshold response (± S.E.). No significant main effect of morph (p = 0.31) or interaction between wavelength and morph was detected (p = 0.94). **(B) Relationship between retinal threshold response and wavelength across *O*. *pumilio* morphs.** Points indicate the mean threshold response (± S.E.). A statistically significant interaction between wavelength and morph was found. Asterisks indicate that Popa morphs have significantly lower thresholds at 450nm and 550nm than Cemetery morphs.

**Fig 9:**
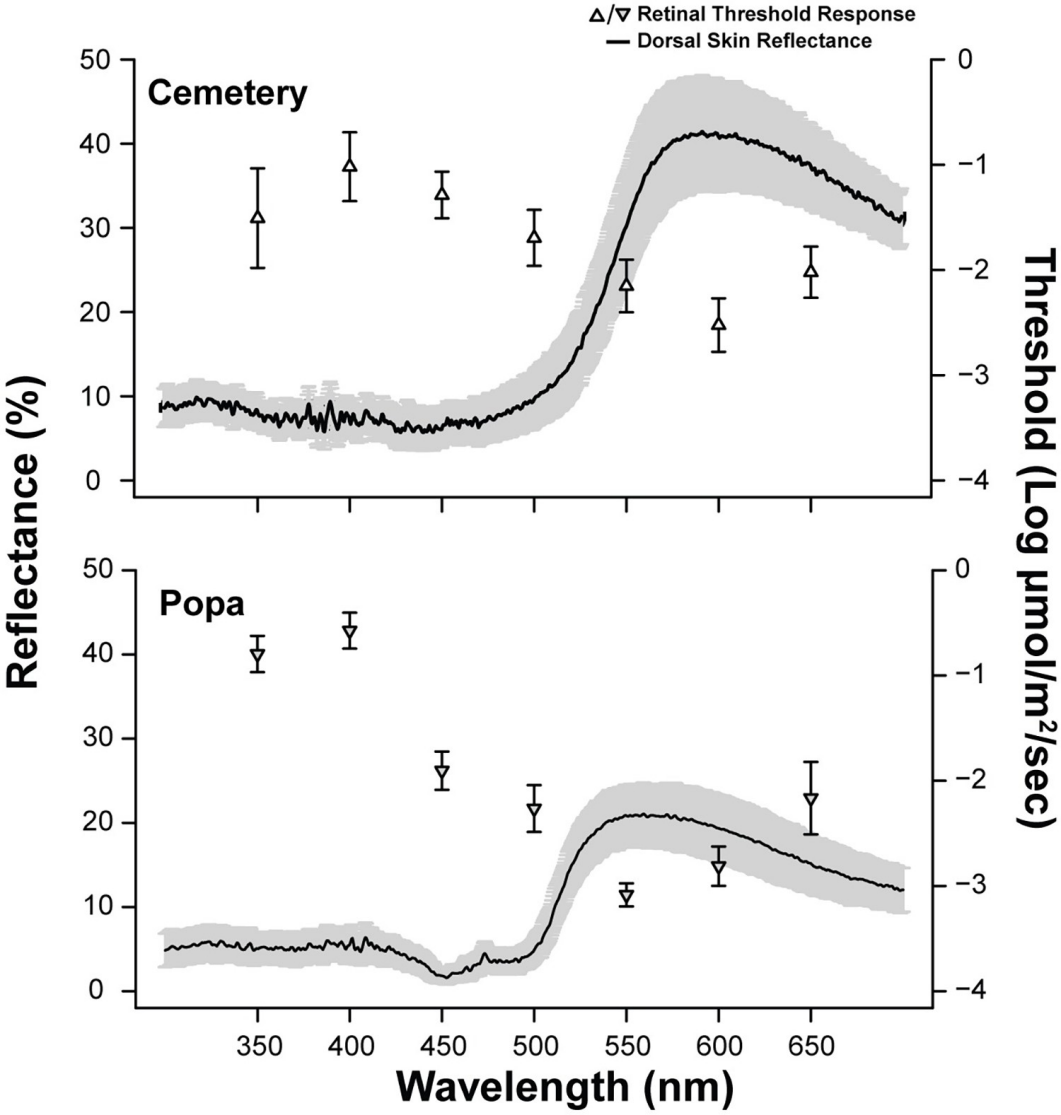
Retinal tuning curves of *O*. *pumilio*, Popa and Cemetery morphs, plotted along with the dorsal reflectance curves. The error bars on the tuning curve points and the shading around the reflectance curve both indicate ± standard error.

Previous data suggest that spectral content is important to mediating behavior in the aposematic species [10,11,37]. In comparison of aposematic tuning curves only, there was a significant main effect of species, where *D*. *tinctorius* had higher retinal thresholds across the spectrum (F[1,178] = 6.84; p < 0.01; Fig 7). However, there was no significant interaction between species and wavelength (p = 0.10). Comparisons within species, but between morphs, indicated that the retinal thresholds of *D*. *tinctorius* morphs did not significantly differ from one another (F [6, 59] = 0.39; p = 0.94; Fig 8A). Conversely, a significant interaction between wavelength and morph was found for *O*. *pumilio* morphs (F [6, 105] = 24; p = 0.04; Fig 8B), where Popa morphs had significantly lower retinal thresholds at 450nm and 550nm. When dorsal reflectance curves were plotted onto the retinal tuning curves of the *O*. *pumilio* morphs, data indicated that the wavelength of peak dorsal reflectance covaried with retinal sensitivity (Fig 9). This suggests that the retinae of *O*. *pumilio* morphs are most sensitive to the wavelengths of light reflected from their dorsal surface. The sample sizes for some species are much smaller for that of *O*. *pumilio*, it is possible that we could have failed to statistically detect covariation between spectral thresholds and spectral reflectance. Therefore, we have included supplemental figures showing the relationship between retinal threshold and dorsal reflectance for all the species used in this study (S11–S16 Figs).

## Discussion

Our analysis of frog skin reflectance data supported the classification of frogs into aposematic, intermediate, and cryptic categories, correlated to their defense strategies. While we did not measure toxicity in the present study, our results complemented studies that have correlated body reflectance and toxicity [21,26]. In particular, Summers et al. (2001), showed a striking positive linear correlation between toxin concentration and reflectance across Dendrobatidae [21]. In that study human observer-based reflectance scores and random pixel sampling by a computer program were used to measure reflectance [21]. Our use of spectrometer measurements and scaled body reflectance confirmed their results: reflectances of *D*. *tinctorius* and *O*. *pumilio* were much higher than that of *A*. *talamancae*, and the reflectance of *P*. *lugubris* fell in the middle (*S*. *flotator* and *C*. *panamansis* were not included in the Summers et al. analysis). Previous studies have attempted to classify this radiation of diurnal frogs in a binary fashion, cryptic versus aposematic/conspicuous coloration [29,62–64]. However, our findings support the addition of an intermediate categorization [42].

We did not find any differences between the retinal thresholds of males and females of any of our species of interest. However, these data should be considered carefully due to low sample sizes and varying reproductive states. In the túngara frog (*Physalaemus pustulosis*), males and females show different ERG responses to white light, where hCG treated females have lower threshold responses [52]. This suggests that the retinae of some species are sensitive to the influence of sex steroids [53]. This phenomenon also manifests behaviorally, where hormonally primed females have more sensitive visual responses [65]. This could potentially play a role in mate choice behavior. While we failed to uncover any retinal variation relating to sex for the species in this study, this could be a lucrative area of research for the future especially given that male and female *O*. *pumilio* show differences in morph specific mate choice behavior [10,11,66].

One thing that should be noted is that our frog species differed in size (SVL and weight), however we have previously found that eye size alone does not predict retinal sensitivity [6]. In fact, our smallest species *S*. *flotator* showed one of the lowest tuning curves, which is the opposite of what we would expect if sensitivity was due to eye aperture alone. Another interesting finding of the current study is that the retinae of aposematic species, *D*. *tinctorius* and *O*. *pumilio*, were less sensitive across the wavelength spectrum than their cryptic or intermediate counterparts. Behaviorally, highly reflective morphs or species are slower to respond to predator presence than are less reflective morphs or species [62,64]. It has been hypothesized that this is due to the presences of high concentrations of toxins in their skin, thereby reducing their risk of predation [62,64]. We found that the retinae of aposematic species required a higher intensity of light to respond, which could contribute to the mechanisms of their latency to react to predator presence. It is possible that the highly sensitive retinae of cryptic and intermediate species is more energetically costly to maintain [3] and that selection for such a costly trait in species that have the added protection of toxicity could be low. This raises an interesting question about an uninvestigated tradeoff between retinal sensitivity and toxicity in aposematic frogs.

In *O*. *pumilio*, and likely in most diurnal frog species, the eye’s lens serves as a powerful UV filter, which is thought to reduce the risk of UV damage to the retina and chromatic aberration [67]. Despite the potential hazards and difficulties of transducing such a high energy waveform many vertebrates do utilize UV vision [68]. Even *O*. *pumilio* behaviorally respond to UV light stimuli [69], and as we found in this current study their retinae are capable of responding to high intensities of UV light. We also found that several species have reflectance peaks in the UV portion of the spectrum, suggesting that these areas of UV reflectance could serve as signaling mechanisms within species or to predators. While avian predators possess UV sensitive visual pigments, frogs lack these proteins [70]. In fact, there is a loss of the SWS-2 opsin in diurnal frog species, biasing diurnal frog vision to longer wavelengths of light [42,43,71]. However, diurnal frogs still possess SWS-1 cones with peak absorbances of 466nm, in *O*. *pumilio* [20]. The absorbance curves of the SWS-1 cones does extend into the UV spectrum, albeit to a lesser degree than is seen at the 466nm peak [20]. Therefore, UV vision in diurnal frogs could be mediated through the tail end of SWS-1 cone sensitivity. Be aware that UV stimuli elicited the highest threshold recordings, meaning high intensities of UV light are required to show a response by the bipolar cells. This is likely due to low absorbance of UV light by the photoreceptors and that the lens filters out ~96% of UV light [20,67]. Future work should focus on downstream retinal signaling and the optic tectum to tease out if these responses can be seen beyond the bipolar cells.

The ERG waveforms of both *D*. *tinctorius* and *O*. *pumilio* in response to UV stimuli exhibited an a-wave followed by a b-wave. However, the a-wave for both species in response to high intensity 350nm stimuli were markedly large and appeared double peaked. We explain this by the differential contributions of rods and cone types to ERG waveforms that can have effects on the waveform shape, particularly the a-wave [55,72]. For example, in generalized or standard clinical protocols with humans, dark adapted rod responses to low amplitude white light flashes exhibit little to no a-wave, but still prominent b-wave (from bipolar cell activity) [72]. In contrast high intensity flashes that stimulate both rods and cones can elicit a large amplitude a-wave [72]. Different photoreceptor contributions are likely not just limited to changes in flash amplitude, however, as changes in flash wavelength could elicit responses from different photoreceptor populations, generating wavelength dependent ERGs. The phenomenon of wavelength dependent ERG responses (voltage trace shapes) has been shown in Zebrafish [73]. For example, flashes of 360 nm (UV) and 580 nm to zebrafish in early stages of development elicit large and small amplitude a-waves, respectively [73]. In mice, ERG responses to UV stimuli show large a-wave components in a fully dark-adapted retina, or scotopic ERG, suggesting that rods could play a role in transducing UV stimuli [74,75]. If we consider the visual pigment absorbances reported for *O*. *pumilio*, all (Rh, SWS-1, MWS, LWS) show a small amount of absorbance at 350nm, with SWS-1 having the highest percentage [20]. Therefore, we suggest that high intensities of UV light stimuli could stimulate multiple photoreceptors to produce the robust a-wave response we recorded.

Our dataset also has interesting implications for phylogenetic inference. Both cryptic species, *A*. *talamancae* and *S*. *flotator*, and intermediate species *C*. *panamansis* are basally located on the Dendrobatid phylogenetic tree [16]. This suggests that the cryptic coloration and retinae sensitive to broadband light stimuli are plesiomorphic and conspicuous coloration and aposematism are an apomorphic in this clade. However, intermediate species *P*. *lugubris* is nested within the aposematic species and is more closely related to *D*. *tinctorius* and *O*. *pumilio* [15,16]. Therefore, our data generate a hypothesis for a reversal to a more ancestral like state in *P*. *lugubris*, where toxin load decreases [25], reflectance decreases, and retinal sensitivity increases. Furthering the phylogenetic implications of our study is the case of *O*. *pumilio*, where we observed that Popa morphs are significantly more sensitive to 450nm and 550nm light stimuli. Popa morphs also had peak dorsal reflectances of 560nm, which corresponds with the lowest wavelength threshold we detected for their retinae (550nm). Cemetery morphs also exhibit a peak in dorsal reflectance (642nm) that is near the most sensitive portion of the retinal tuning curve (600nm). Taken together, this is consistent with the matched filter hypothesis where the visual signal spectrum matches visual sensitivity [76,77]. Based on the literature what could cause this shift? Siddiqi et al., 2004 showed that the peak LWS cone absorbance is 560nm, therefore our findings suggest a large contribution of LWS cones for both morphs. However, this does not rule out the contribution of photoreceptor types, for example the FWHM of the cemetery dorsal reflectance peak extends from 543nm-740.72nm which could activate SWS and MWS cones to a lesser degree [20]. Shifts in sensitivity could be due to a shift in opsin expression [43,44], ocular media transmittance [78], or even modulation at the bipolar cell layer [46]. However, we cannot address the mechanism of the shift using a gross electrophysiological measure like the ERG.

From an evolutionary perspective, the *O*. *pumilio* results could imply there could be more variance in physiological sensitivity in *O*. *pumilio* due to their phenotypic radiation that produced distinct color morphs across island populations [48]. Indeed, this species shows visual preference for their own color morphs [10,11], which we suggest could be mediated by an increase in retinal sensitivity to portions of the light spectrum (as we observed in the Popa morph). While not the focus of the present study, a comparison of retinal physiologies between mainland and island populations of *O*. *pumilio* could elucidate if a sensory exploitation contributed to phenotypic divergence, i.e. if retinal physiology shifted prior to reflectance changes [79].

In summary, we found that frogs that exhibit distinctive reflective strategies also show differences in their retinal sensitivity. However, the degree of wavelength specificity did not seem to change until comparisons were made within *O*. *pumilio*. Nevertheless, using a gross measure of retinal function, we were able to detect species level differences in spectral sensitivity. We also found that the diurnal frog retinae can respond to wavelengths spanning the visible light spectrum, and into the UV. Future studies should focus on the cellular and evolutionary mechanisms that drive retinal sensitivity and diversity within this clade.

## Supporting information

S1 Fig A schematic illustrating quantification of spectral reflectance curves. Peak was defined as the wavelength (nm) with maximum reflectance. FWHM was calculated as the distance between the wavelengths (nm, bandwidth) on either side of the peak where half of the maximum reflectance occurred. The AUC measurement was the integral of the reflectance curve. The average reflectance (%) is the mean of all reflectance measurements across the entire spectrum.
(TIF)

S2 Fig A typical ERG trace illustrating the hyperpolarization of photoreceptors, the a-wave, followed by the depolarization of bipolar cells, the b-wave. (TIF)

S3 Fig ERG data for *D*. *tinctorius* (Pat.). **(A)** Each graph shows example raw ERG traces from a single individual at a specific wavelength (nm) which is labeled in the lower-right corner of each graph. The three traces within each graph show ERG responses to pre-threshold (blue), mid-curve (yellow), and near-saturation (red) light intensities. The arrows on the 500nm graph show typical a-wave and b-wave responses; in other graphs, these responses are unlabeled but can be seen. **(B)** Each graph displays the V-log(I) curves generated from relative b-wave responses (triangles) across light intensities for the same individual shown in figure A_1_. Again, wavelengths (nm) are labeled in the lower-right corner. The outlined, enlarged triangles correspond to the pre-threshold (blue), mid-curve (yellow), and near-saturation (red) responses displayed in Figure A_1_. On the 500nm graph, the light intensity (μmol/m^2^/sec) at which the dotted and solid lines meet is considered the 10% threshold response; this response is unlabeled in other graphs but was calculated for all wavelengths. **(C)** Each graph displays the V-log(I) curves generated from mean (± S.E.) relative b-wave responses (triangles) across light intensities for all *D*. *tinctorius* (Pat.) specimens. The wavelengths (nm) are labeled in the lower-right corner of each graph.
(TIF)

S4 Fig ERG data for *O*. *pumilio* (Cem.). **(A)** Each graph shows example raw ERG traces from a single individual at a specific wavelength (nm) which is labeled in the lower-right corner of each graph. The three traces within each graph show ERG responses to pre-threshold (blue), mid-curve (yellow), and near-saturation (red) light intensities. The arrows on the 500nm graph show typical a-wave and b-wave responses; in other graphs, these responses are unlabeled but can be seen. **(B)** Each graph displays the V-log(I) curves generated from relative b-wave responses (upside-down triangles) across light intensities for the same individual shown in figure A_1_. Again, wavelengths (nm) are labeled in the lower-right corner. The outlined, enlarged upside-down triangles correspond to the pre-threshold (blue), mid-curve (yellow), and near-saturation (red) responses displayed in Figure A_1_. On the 500nm graph, the light intensity (μmol/m^2^/sec) at which the dotted and solid lines meet is considered the 10% threshold response; this response is unlabeled in other graphs but was calculated for all wavelengths. **(C)** Each graph displays the V-log(I) curves generated from mean (± S.E.) relative b-wave responses (upside-down triangles) across light intensities for all *O*. *pumilio* (Cem.) specimens. The wavelengths (nm) are labeled in the lower-right corner of each graph.
(TIF)

S5 Fig ERG data for *O*. *pumilio* (Pop.). **(A)** Each graph shows example raw ERG traces from a single individual at a specific wavelength (nm) which is labeled in the lower-right corner of each graph. The three traces within each graph show ERG responses to pre-threshold (blue), mid-curve (yellow), and near-saturation (red) light intensities. The arrows on the 500nm graph show typical a-wave and b-wave responses; in other graphs, these responses are unlabeled but can be seen. **(B)** Each graph displays the V-log(I) curves generated from relative b-wave responses (upside-down triangles) across light intensities for the same individual shown in figure A_1_. Again, wavelengths (nm) are labeled in the lower-right corner. The outlined, enlarged upside-down triangles correspond to the pre-threshold (blue), mid-curve (yellow), and near-saturation (red) responses displayed in Figure A_1_. On the 500nm graph, the light intensity (μmol/m^2^/sec) at which the dotted and solid lines meet is considered the 10% threshold response; this response is unlabeled in other graphs but was calculated for all wavelengths. **(C)** Each graph displays the V-log(I) curves generated from mean (± S.E.) relative b-wave responses (upside-down triangles) across light intensities for all *O*. *pumilio* (Pop.) specimens. The wavelengths (nm) are labeled in the lower-right corner of each graph.
(TIF)

S6 Fig ERG data for *C*. *panamansis*. **(A)** Each graph shows example raw ERG traces from a single individual at a specific wavelength (nm) which is labeled in the lower-right corner of each graph. The three traces within each graph show ERG responses to pre-threshold (blue), mid-curve (yellow), and near-saturation (red) light intensities. The arrows on the 500nm graph show typical a-wave and b-wave responses; in other graphs, these responses are unlabeled but can be seen. **(B)** Each graph displays the V-log(I) curves generated from relative b-wave responses (circles) across light intensities for the same individual shown in figure A_1_. Again, wavelengths (nm) are labeled in the lower-right corner. The outlined, enlarged circles correspond to the pre-threshold (blue), mid-curve (yellow), and near-saturation (red) responses displayed in Figure A_1_. On the 500nm graph, the light intensity (μmol/m^2^/sec) at which the dotted and solid lines meet is considered the 10% threshold response; this response is unlabeled in other graphs but was calculated for all wavelengths. **(C)** Each graph displays the V-log(I) curves generated from mean (± S.E.) relative b-wave responses (circles) across light intensities for all *C*. *panamansis* specimens. The wavelengths (nm) are labeled in the lower-right corner of each graph.
(TIF)

S7 Fig ERG data for *P*. *lugubris*. **(A)** Each graph shows example raw ERG traces from a single individual at a specific wavelength (nm) which is labeled in the lower-right corner of each graph. The three traces within each graph show ERG responses to pre-threshold (blue), mid-curve (yellow), and near-saturation (red) light intensities. The arrows on the 500nm graph show typical a-wave and b-wave responses; in other graphs, these responses are unlabeled but can be seen. **(B)** Each graph displays the V-log(I) curves generated from relative b-wave responses (X/circles) across light intensities for the same individual shown in figure A_1_. Again, wavelengths (nm) are labeled in the lower-right corner. The outlined, enlarged X/circles correspond to the pre-threshold (blue), mid-curve (yellow), and near-saturation (red) responses displayed in Figure A_1_. On the 500nm graph, the light intensity (μmol/m^2^/sec) at which the dotted and solid lines meet is considered the 10% threshold response; this response is unlabeled in other graphs but was calculated for all wavelengths. **(C)** Each graph displays the V-log(I) curves generated from mean (± S.E.) relative b-wave responses (X/circles) across light intensities for all *P*. *lugubris* specimens. The wavelengths (nm) are labeled in the lower-right corner of each graph.
(TIF)

S8 Fig ERG data for *A*. *talamancae*. **(A)** Each graph shows example raw ERG traces from a single individual at a specific wavelength (nm) which is labeled in the lower-right corner of each graph. The three traces within each graph show ERG responses to pre-threshold (blue), mid-curve (yellow), and near-saturation (red) light intensities. The arrows on the 500nm graph show typical a-wave and b-wave responses; in other graphs, these responses are unlabeled but can be seen. **(B)** Each graph displays the V-log(I) curves generated from relative b-wave responses (squares) across light intensities for the same individual shown in figure A_1_. Again, wavelengths (nm) are labeled in the lower-right corner. The outlined, enlarged squares correspond to the pre-threshold (blue), mid-curve (yellow), and near-saturation (red) responses displayed in Figure A_1_. On the 500nm graph, the light intensity (μmol/m^2^/sec) at which the dotted and solid lines meet is considered the 10% threshold response; this response is unlabeled in other graphs but was calculated for all wavelengths. **(C)** Each graph displays the V-log(I) curves generated from mean (± S.E.) relative b-wave responses (squares) across light intensities for all *A*. *talamancae* specimens. The wavelengths (nm) are labeled in the lower-right corner of each graph.
(TIF)

S9 Fig ERG data for *S*. *flotator*. **(A)** Each graph shows example raw ERG traces from a single individual at a specific wavelength (nm) which is labeled in the lower-right corner of each graph. The three traces within each graph show ERG responses to pre-threshold (blue), mid-curve (yellow), and near-saturation (red) light intensities. The arrows on the 500nm graph show typical a-wave and b-wave responses; in other graphs, these responses are unlabeled but can be seen. **(B)** Each graph displays the V-log(I) curves generated from relative b-wave responses (cross/squares) across light intensities for the same individual shown in figure A_1_. Again, wavelengths (nm) are labeled in the lower-right corner. The outlined, enlarged cross/squares correspond to the pre-threshold (blue), mid-curve (yellow), and near-saturation (red) responses displayed in Figure A_1_. On the 500nm graph, the light intensity (μmol/m^2^/sec) at which the dotted and solid lines meet is considered the 10% threshold response; this response is unlabeled in other graphs but was calculated for all wavelengths. **(C)** Each graph displays the V-log(I) curves generated from mean (± S.E.) relative b-wave responses (cross/squares) across light intensities for all *S*. *flotator* specimens. The wavelengths (nm) are labeled in the lower-right corner of each graph.
(TIF)

S10 Fig Relationship between retinal threshold response and wavelength across species/morphs. Points indicate the mean threshold response (± S.E.). A statistically significant main effect of species was found.
(TIF)

S11 Fig Retinal tuning curve of *D*. *tinctorius* Az. morph plotted along with the dorsal reflectance curves. The error bars on the tuning curve points and the shading around the reflectance curve both indicate ± standard error.
(TIF)

S12 Fig Retinal tuning curve of *D*. *tinctorius* Pat. morph plotted along with the dorsal reflectance curves. The error bars on the tuning curve points and the shading around the reflectance curve both indicate ± standard error.
(TIF)

S13 Fig Retinal tuning curve of *P*. *lugubris* plotted along with the dorsal reflectance curves. The error bars on the tuning curve points and the shading around the reflectance curve both indicate ± standard error.
(TIF)

S14 Fig Retinal tuning curve of *C*, *panamansis* plotted along with the dorsal reflectance curves. The error bars on the tuning curve points and the shading around the reflectance curve both indicate ± standard error.
(TIF)

S15 Fig Retinal tuning curve of *A*. *talamancae* plotted along with the dorsal reflectance curves. The error bars on the tuning curve points and the shading around the reflectance curve both indicate ± standard error.
(TIF)

S16 Fig Retinal tuning curve of *S*. *flotator* plotted along with the dorsal reflectance curves. The error bars on the tuning curve points and the shading around the reflectance curve both indicate ± standard error.
(TIF)

S1 Table A table showing the analysis of reflectance data from spectrometer measurements (300-700nm) for all the species and species morphs used in this study. Reflectance curves were gathered for distinct morphological (dorsal, lateral, axillary, and inguinal) regions for each individual specimen. Additional reflectance curves were measured for subregions where there were obvious spectral differences that could be detected by the human eye (i.e. dorsal black and dorsal blue). The data displayed on this table are the mean measurements (± S.E.).
(DOCX)

S2 Table The results of a pairwise comparison with a Bonferroni correction on the threshold retinal responses of dendrobatid species. *D*. *tinctorius* (*D*. *t*.), *O*. *pumilio* (*O*. *p*.), *C*. *panamansis* (*C*. *p*.), *P*. *lugubris* (*P*. *l*.), *A*. *talamancae* (*A*. *t*.), and *S*. *flotator* (S. f.).
(DOCX)

S3 Table The results of a pairwise comparison with a Bonferroni correction on the threshold retinal responses of dendrobatid species/morphs. *D*. *tinctorius* (*D*. *t*. Az. And Pat.), *O*. *pumilio* (*O*. *p*. Cem. and Pop.), *C*. *panamansis* (*C*. *p*.), *P*. *lugubris* (*P*. *l*.), *A*. *talamancae* (*A*. *t*.), and *S*. *flotator* (S. f.).
(DOCX)

S4 Table The results of one-way ANOVAs between males and females of each species of dendrobatid. *D*. *tinctorius* (*D*. *t*. Az. And Pat.), *O*. *pumilio* (*O*. *p*. Cem. and Pop.), *C*. *panamansis* (*C*. *p*.), *P*. *lugubris* (*P*. *l*.), *A*. *talamancae* (*A*. *t*.), and *S*. *flotator* (S. f.).
(DOCX)
