# Supplementary figures and images for: The relationship between spectral signals and retinal sensitivity in dendrobatid frogs
Source: PLoS One. 2024 Nov 14;19(11):e0312578. doi: 10.1371/journal.pone.0312578 (PMC11563434; doi:10.1371/journal.pone.0312578)

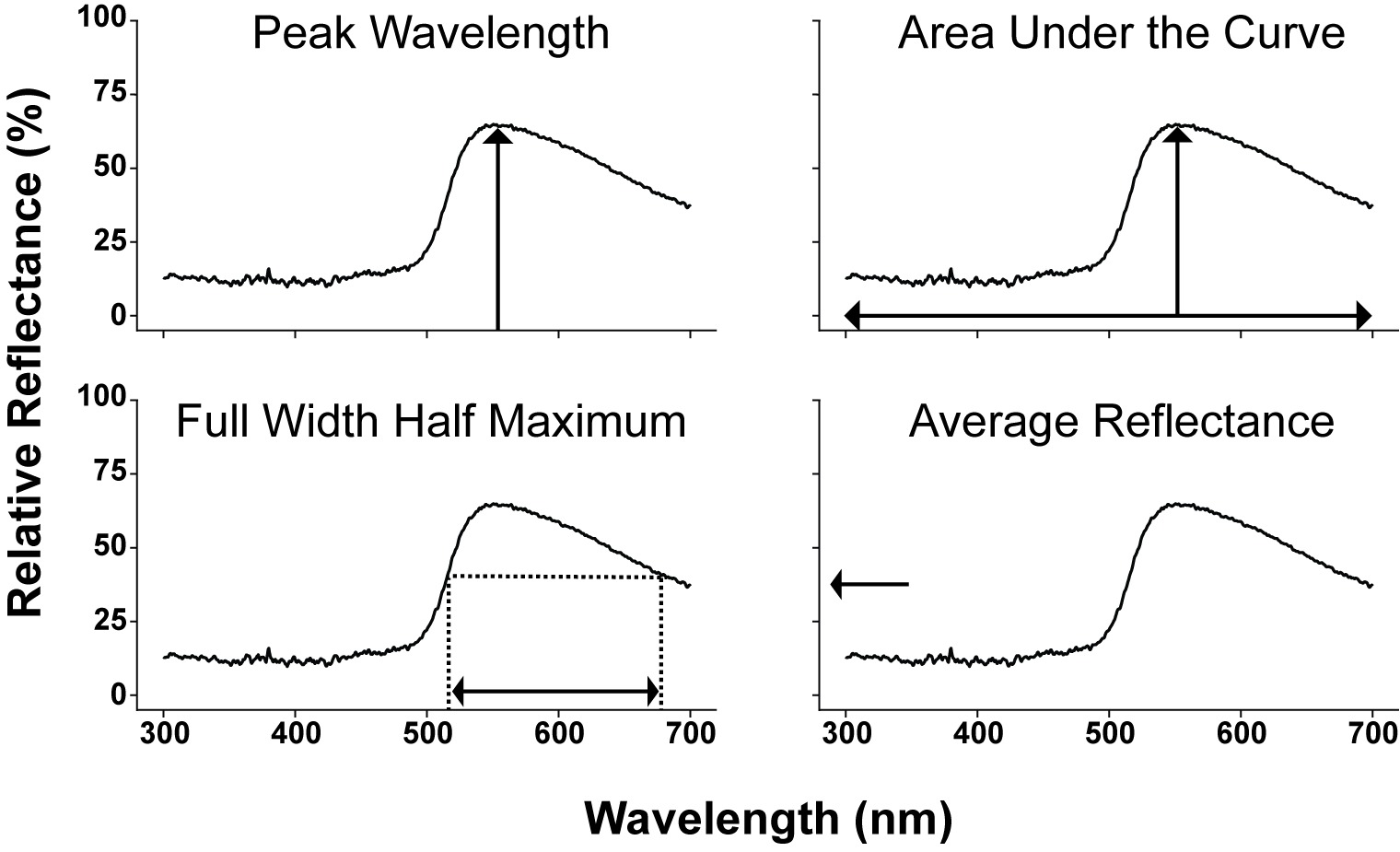

Supplement: S1 Fig — Peak was defined as the wavelength (nm) with maximum reflectance. FWHM was calculated as the distance between the wavelengths (nm, bandwidth) on either side of the peak where half of the maximum reflectance occurred. The AUC measurement was the integral of the reflectance curve. The average reflectance (%) is the mean of all reflectance measurements across the entire spectrum. (TIF) [file pone.0312578.s001.tif]

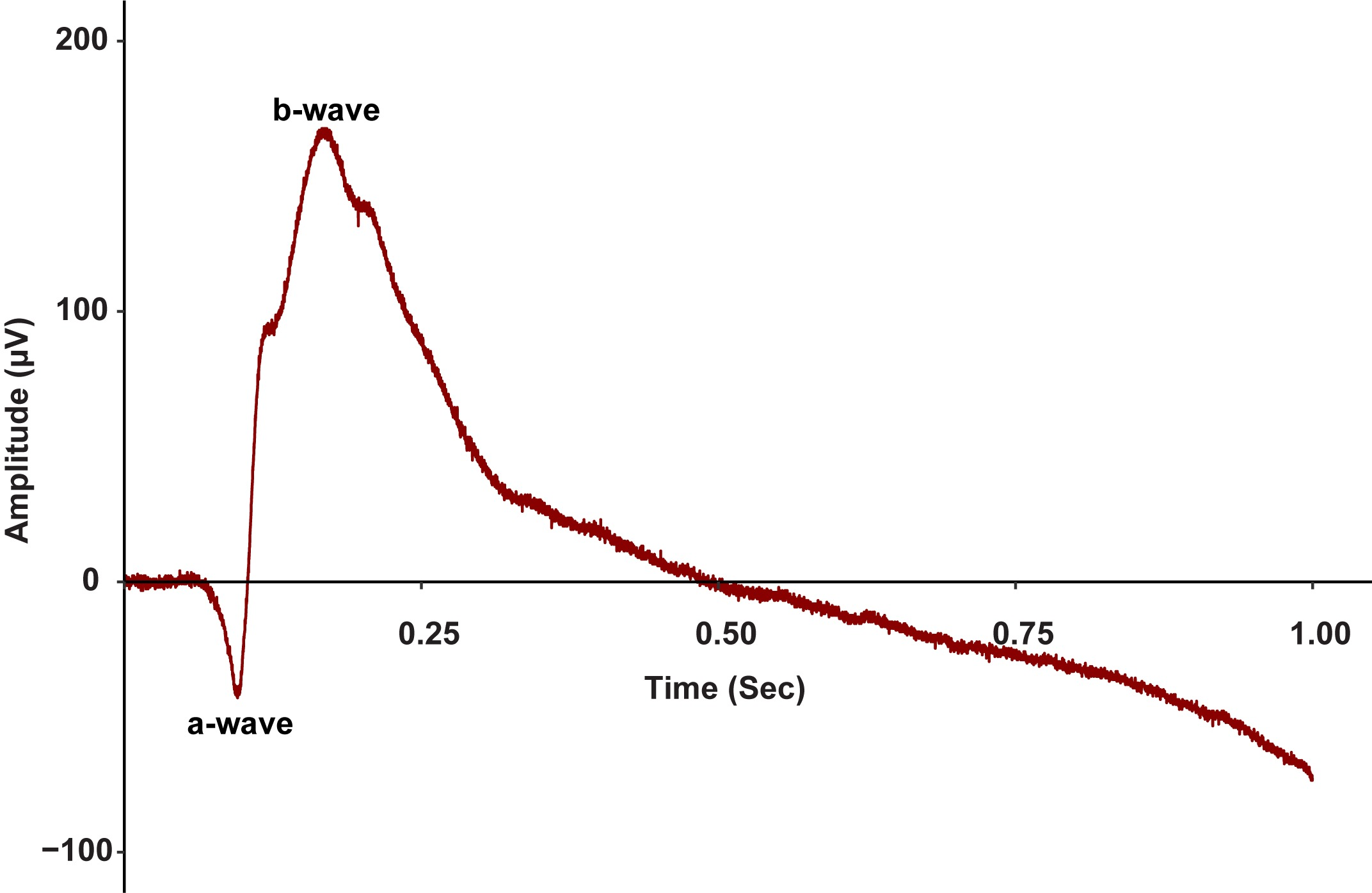

Supplement: S2 Fig — (TIF) [file pone.0312578.s002.tif]

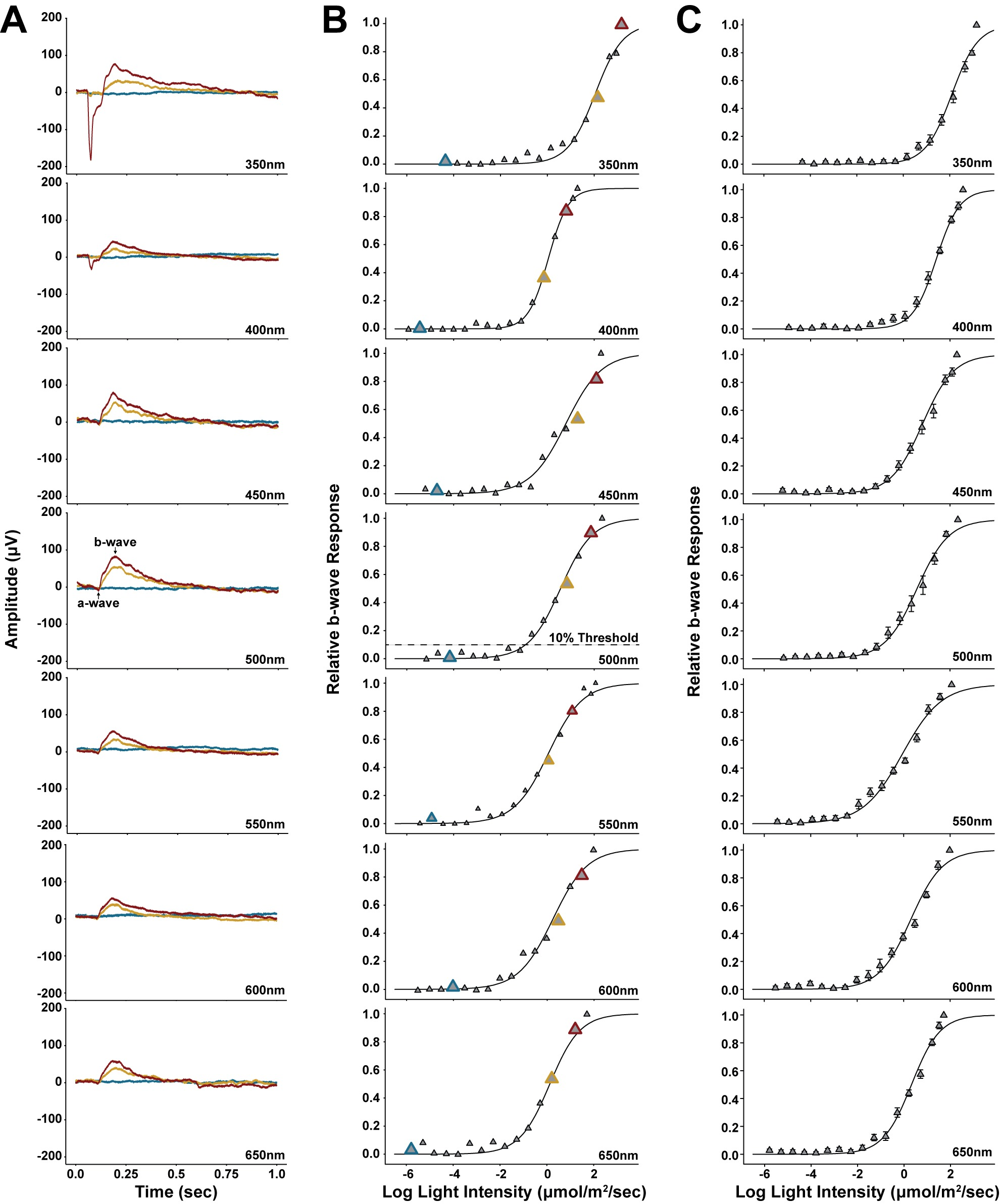

Supplement: S3 Fig — (A) Each graph shows example raw ERG traces from a single individual at a specific wavelength (nm) which is labeled in the lower-right corner of each graph. The three traces within each graph show ERG responses to pre-threshold (blue), mid-curve (yellow), and near-saturation (red) light intensities. The arrows on the 500nm graph show typical a-wave and b-wave responses; in other graphs, these responses are unlabeled but can be seen. (B) Each graph displays the V-log(I) curves generated from relative b-wave responses (triangles) across light intensities for the same individual shown in figure A1. Again, wavelengths (nm) are labeled in the lower-right corner. The outlined, enlarged triangles correspond to the pre-threshold (blue), mid-curve (yellow), and near-saturation (red) responses displayed in Figure A1. On the 500nm graph, the light intensity (μmol/m2/sec) at which the dotted and solid lines meet is considered the 10% threshold response; this response is unlabeled in other graphs but was calculated for all wavelengths. (C) Each graph displays the V-log(I) curves generated from mean (± S.E.) relative b-wave responses (triangles) across light intensities for all D. tinctorius (Pat.) specimens. The wavelengths (nm) are labeled in the lower-right corner of each graph. (TIF) [file pone.0312578.s003.tif]

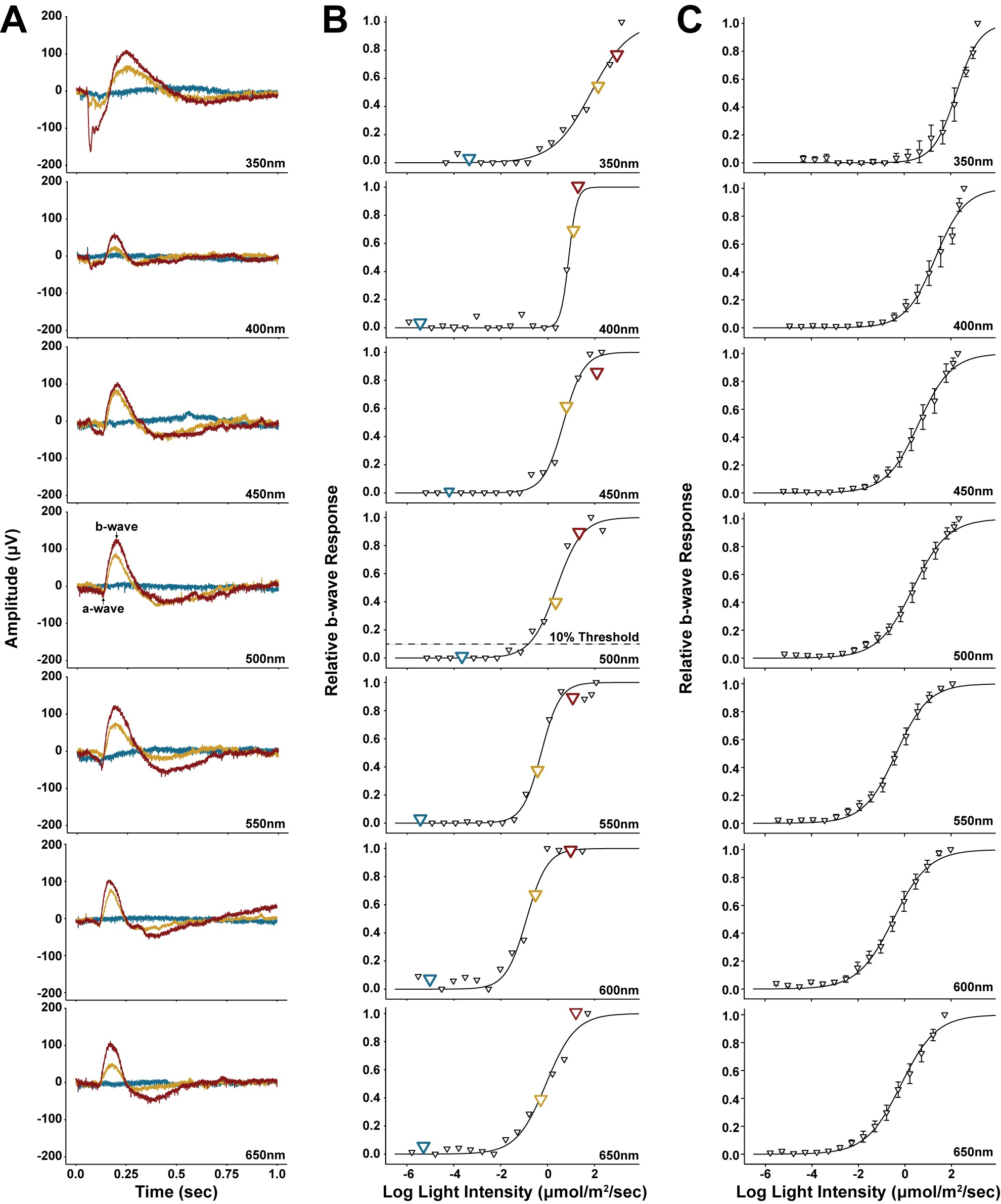

Supplement: S4 Fig — (A) Each graph shows example raw ERG traces from a single individual at a specific wavelength (nm) which is labeled in the lower-right corner of each graph. The three traces within each graph show ERG responses to pre-threshold (blue), mid-curve (yellow), and near-saturation (red) light intensities. The arrows on the 500nm graph show typical a-wave and b-wave responses; in other graphs, these responses are unlabeled but can be seen. (B) Each graph displays the V-log(I) curves generated from relative b-wave responses (upside-down triangles) across light intensities for the same individual shown in figure A1. Again, wavelengths (nm) are labeled in the lower-right corner. The outlined, enlarged upside-down triangles correspond to the pre-threshold (blue), mid-curve (yellow), and near-saturation (red) responses displayed in Figure A1. On the 500nm graph, the light intensity (μmol/m2/sec) at which the dotted and solid lines meet is considered the 10% threshold response; this response is unlabeled in other graphs but was calculated for all wavelengths. (C) Each graph displays the V-log(I) curves generated from mean (± S.E.) relative b-wave responses (upside-down triangles) across light intensities for all O. pumilio (Cem.) specimens. The wavelengths (nm) are labeled in the lower-right corner of each graph. (TIF) [file pone.0312578.s004.tif]

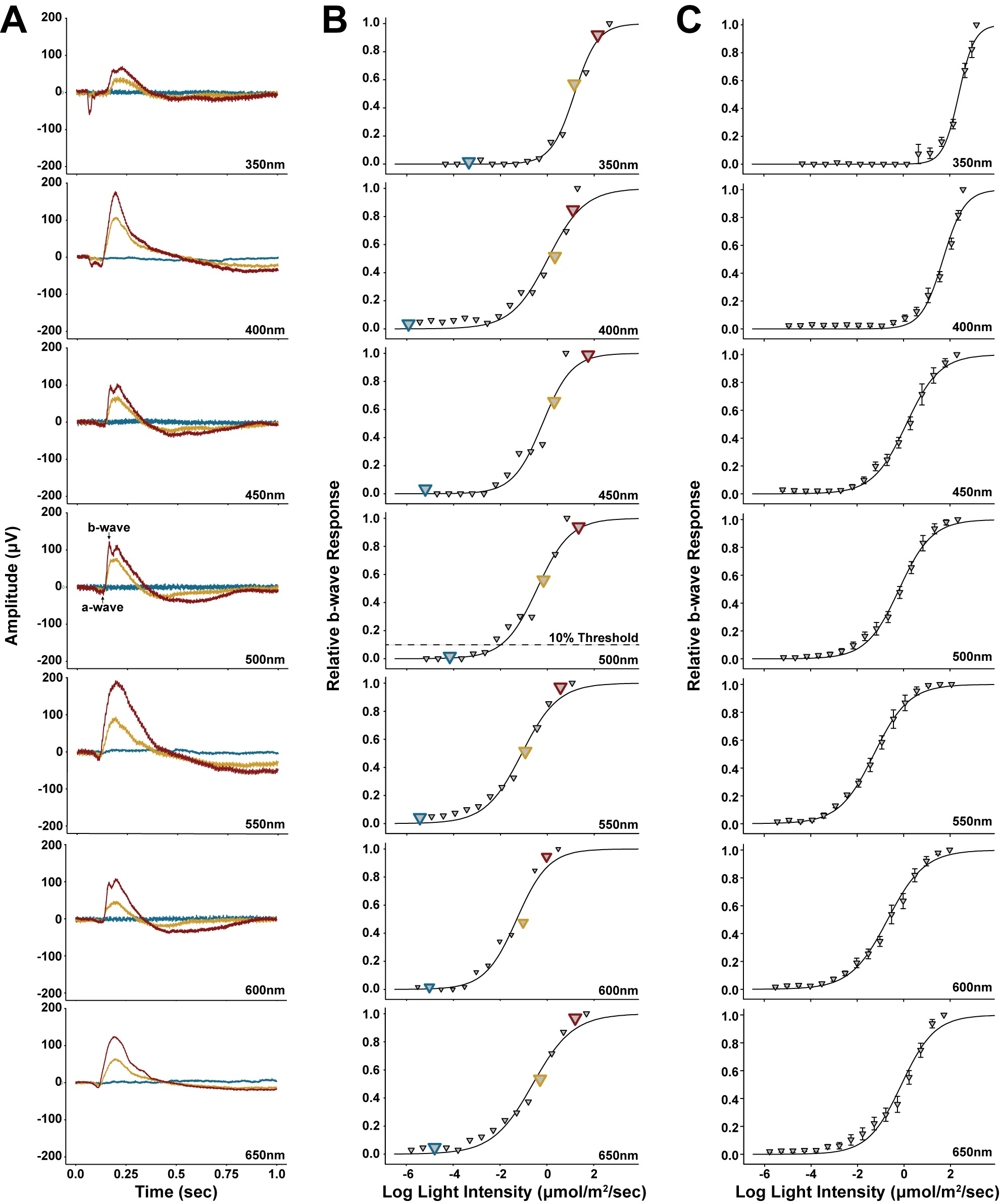

Supplement: S5 Fig — (A) Each graph shows example raw ERG traces from a single individual at a specific wavelength (nm) which is labeled in the lower-right corner of each graph. The three traces within each graph show ERG responses to pre-threshold (blue), mid-curve (yellow), and near-saturation (red) light intensities. The arrows on the 500nm graph show typical a-wave and b-wave responses; in other graphs, these responses are unlabeled but can be seen. (B) Each graph displays the V-log(I) curves generated from relative b-wave responses (upside-down triangles) across light intensities for the same individual shown in figure A1. Again, wavelengths (nm) are labeled in the lower-right corner. The outlined, enlarged upside-down triangles correspond to the pre-threshold (blue), mid-curve (yellow), and near-saturation (red) responses displayed in Figure A1. On the 500nm graph, the light intensity (μmol/m2/sec) at which the dotted and solid lines meet is considered the 10% threshold response; this response is unlabeled in other graphs but was calculated for all wavelengths. (C) Each graph displays the V-log(I) curves generated from mean (± S.E.) relative b-wave responses (upside-down triangles) across light intensities for all O. pumilio (Pop.) specimens. The wavelengths (nm) are labeled in the lower-right corner of each graph. (TIF) [file pone.0312578.s005.tif]

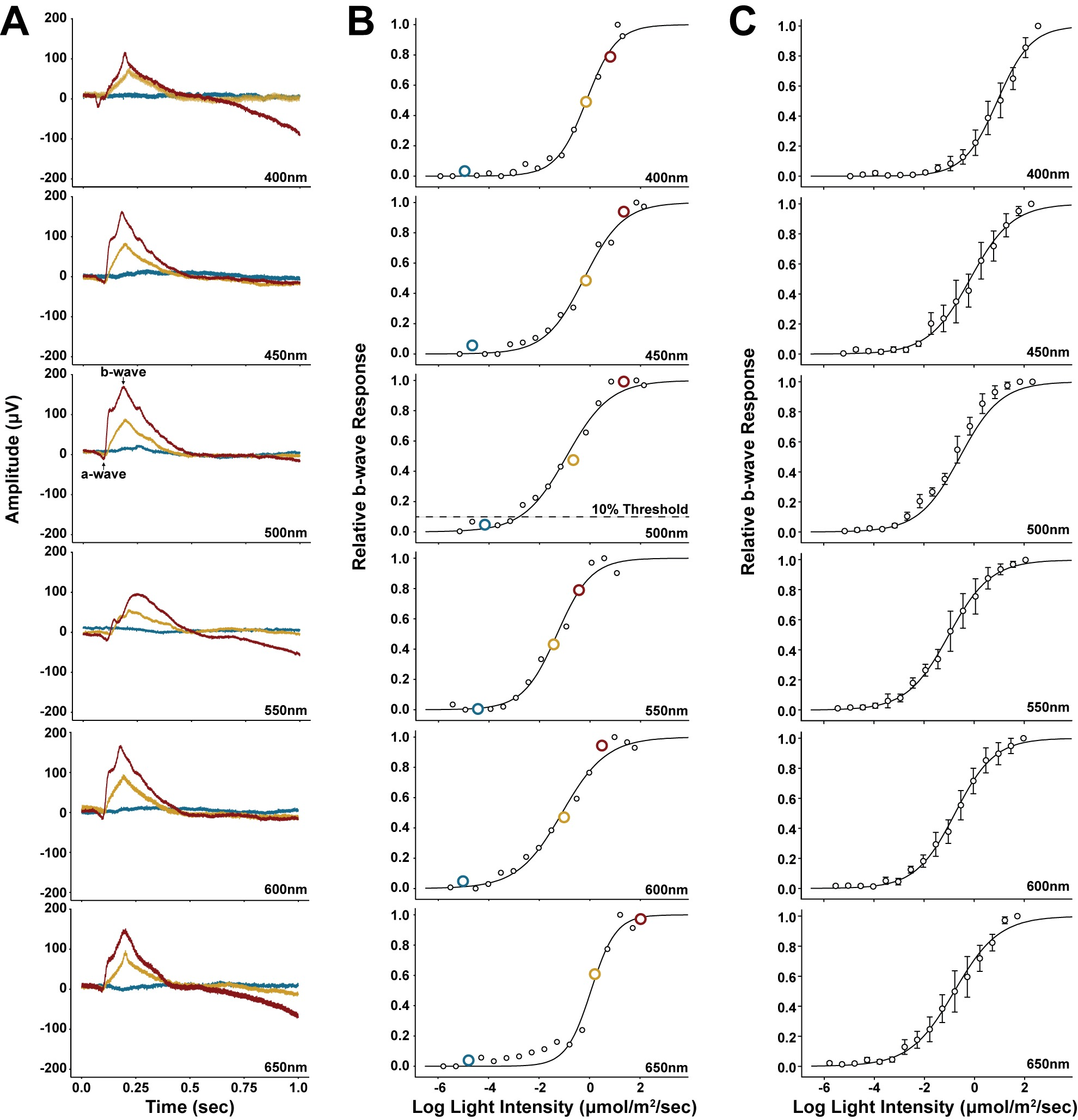

Supplement: S6 Fig — (A) Each graph shows example raw ERG traces from a single individual at a specific wavelength (nm) which is labeled in the lower-right corner of each graph. The three traces within each graph show ERG responses to pre-threshold (blue), mid-curve (yellow), and near-saturation (red) light intensities. The arrows on the 500nm graph show typical a-wave and b-wave responses; in other graphs, these responses are unlabeled but can be seen. (B) Each graph displays the V-log(I) curves generated from relative b-wave responses (circles) across light intensities for the same individual shown in figure A1. Again, wavelengths (nm) are labeled in the lower-right corner. The outlined, enlarged circles correspond to the pre-threshold (blue), mid-curve (yellow), and near-saturation (red) responses displayed in Figure A1. On the 500nm graph, the light intensity (μmol/m2/sec) at which the dotted and solid lines meet is considered the 10% threshold response; this response is unlabeled in other graphs but was calculated for all wavelengths. (C) Each graph displays the V-log(I) curves generated from mean (± S.E.) relative b-wave responses (circles) across light intensities for all C. panamansis specimens. The wavelengths (nm) are labeled in the lower-right corner of each graph. (TIF) [file pone.0312578.s006.tif]

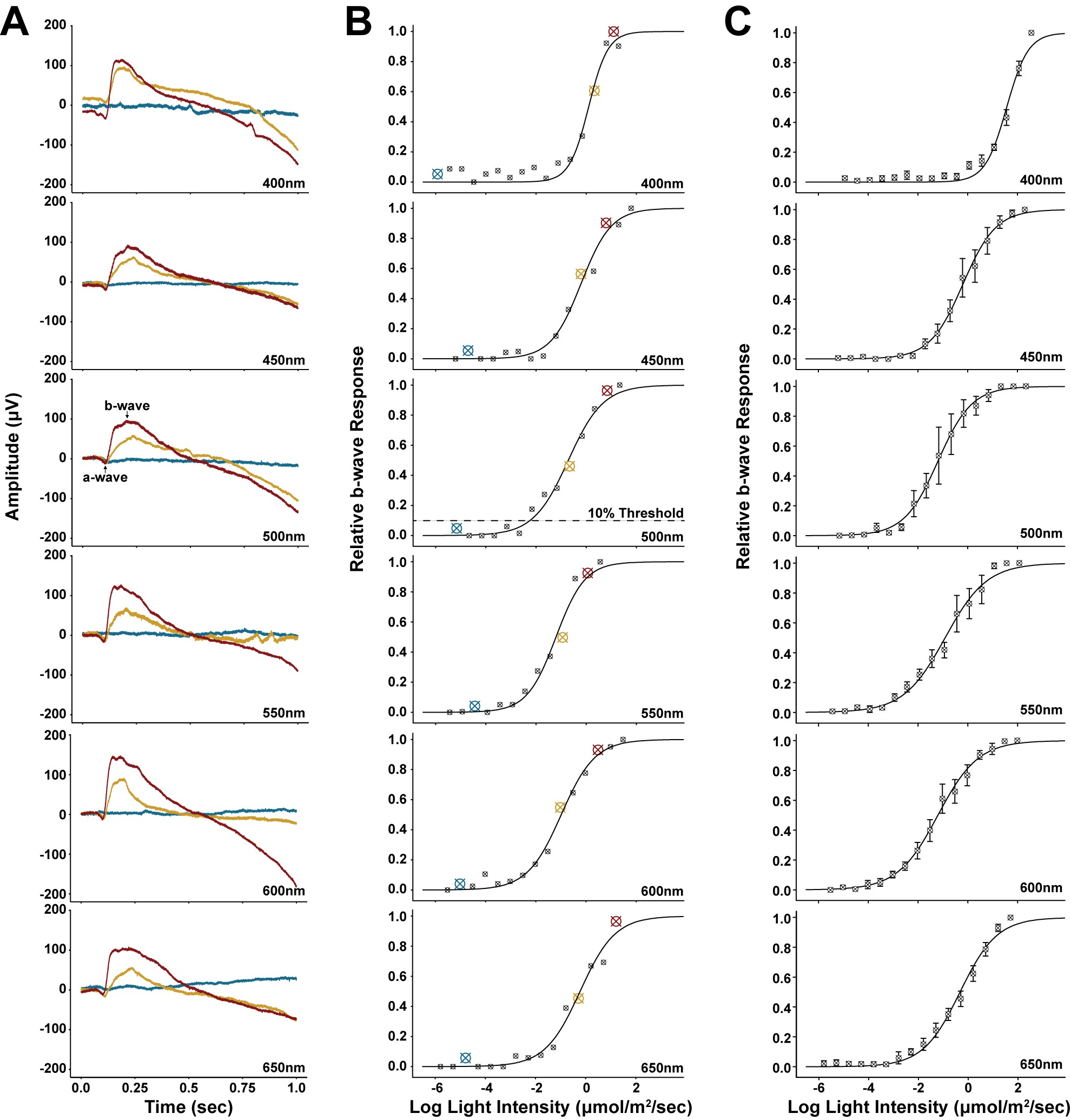

Supplement: S7 Fig — (A) Each graph shows example raw ERG traces from a single individual at a specific wavelength (nm) which is labeled in the lower-right corner of each graph. The three traces within each graph show ERG responses to pre-threshold (blue), mid-curve (yellow), and near-saturation (red) light intensities. The arrows on the 500nm graph show typical a-wave and b-wave responses; in other graphs, these responses are unlabeled but can be seen. (B) Each graph displays the V-log(I) curves generated from relative b-wave responses (X/circles) across light intensities for the same individual shown in figure A1. Again, wavelengths (nm) are labeled in the lower-right corner. The outlined, enlarged X/circles correspond to the pre-threshold (blue), mid-curve (yellow), and near-saturation (red) responses displayed in Figure A1. On the 500nm graph, the light intensity (μmol/m2/sec) at which the dotted and solid lines meet is considered the 10% threshold response; this response is unlabeled in other graphs but was calculated for all wavelengths. (C) Each graph displays the V-log(I) curves generated from mean (± S.E.) relative b-wave responses (X/circles) across light intensities for all P. lugubris specimens. The wavelengths (nm) are labeled in the lower-right corner of each graph. (TIF) [file pone.0312578.s007.tif]

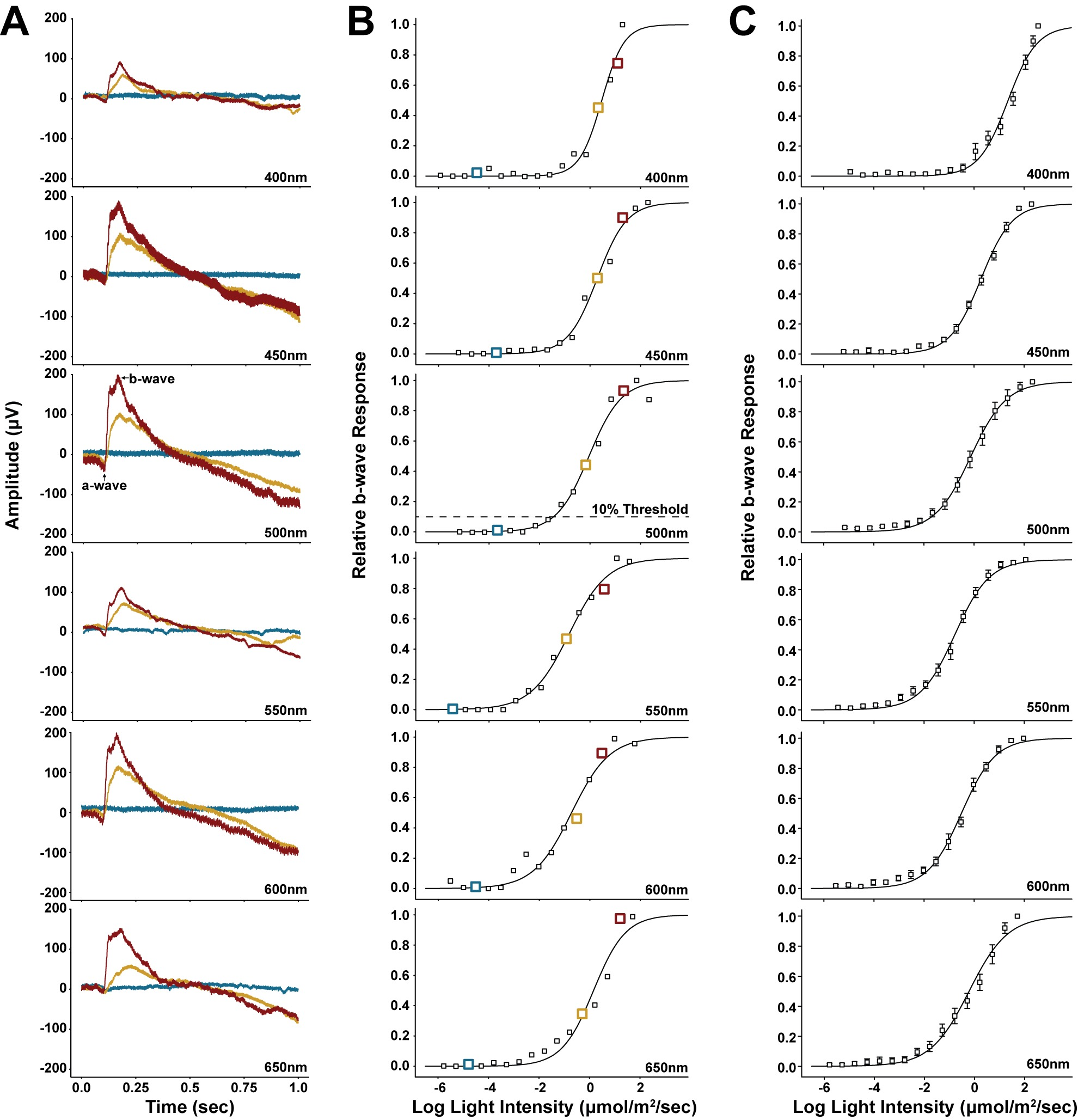

Supplement: S8 Fig — (A) Each graph shows example raw ERG traces from a single individual at a specific wavelength (nm) which is labeled in the lower-right corner of each graph. The three traces within each graph show ERG responses to pre-threshold (blue), mid-curve (yellow), and near-saturation (red) light intensities. The arrows on the 500nm graph show typical a-wave and b-wave responses; in other graphs, these responses are unlabeled but can be seen. (B) Each graph displays the V-log(I) curves generated from relative b-wave responses (squares) across light intensities for the same individual shown in figure A1. Again, wavelengths (nm) are labeled in the lower-right corner. The outlined, enlarged squares correspond to the pre-threshold (blue), mid-curve (yellow), and near-saturation (red) responses displayed in Figure A1. On the 500nm graph, the light intensity (μmol/m2/sec) at which the dotted and solid lines meet is considered the 10% threshold response; this response is unlabeled in other graphs but was calculated for all wavelengths. (C) Each graph displays the V-log(I) curves generated from mean (± S.E.) relative b-wave responses (squares) across light intensities for all A. talamancae specimens. The wavelengths (nm) are labeled in the lower-right corner of each graph. (TIF) [file pone.0312578.s008.tif]

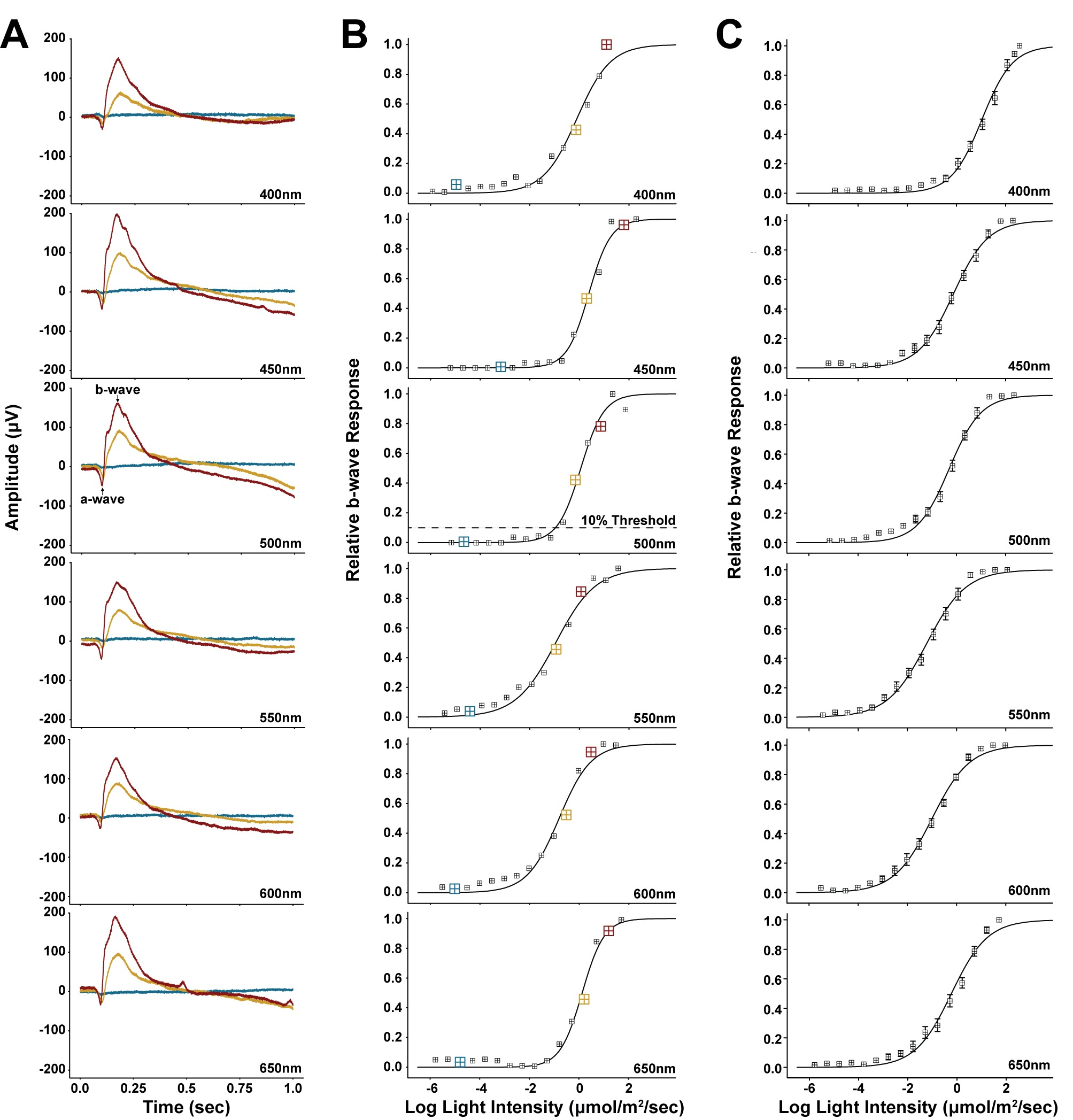

Supplement: S9 Fig — (A) Each graph shows example raw ERG traces from a single individual at a specific wavelength (nm) which is labeled in the lower-right corner of each graph. The three traces within each graph show ERG responses to pre-threshold (blue), mid-curve (yellow), and near-saturation (red) light intensities. The arrows on the 500nm graph show typical a-wave and b-wave responses; in other graphs, these responses are unlabeled but can be seen. (B) Each graph displays the V-log(I) curves generated from relative b-wave responses (cross/squares) across light intensities for the same individual shown in figure A1. Again, wavelengths (nm) are labeled in the lower-right corner. The outlined, enlarged cross/squares correspond to the pre-threshold (blue), mid-curve (yellow), and near-saturation (red) responses displayed in Figure A1. On the 500nm graph, the light intensity (μmol/m2/sec) at which the dotted and solid lines meet is considered the 10% threshold response; this response is unlabeled in other graphs but was calculated for all wavelengths. (C) Each graph displays the V-log(I) curves generated from mean (± S.E.) relative b-wave responses (cross/squares) across light intensities for all S. flotator specimens. The wavelengths (nm) are labeled in the lower-right corner of each graph. (TIF) [file pone.0312578.s009.tif]

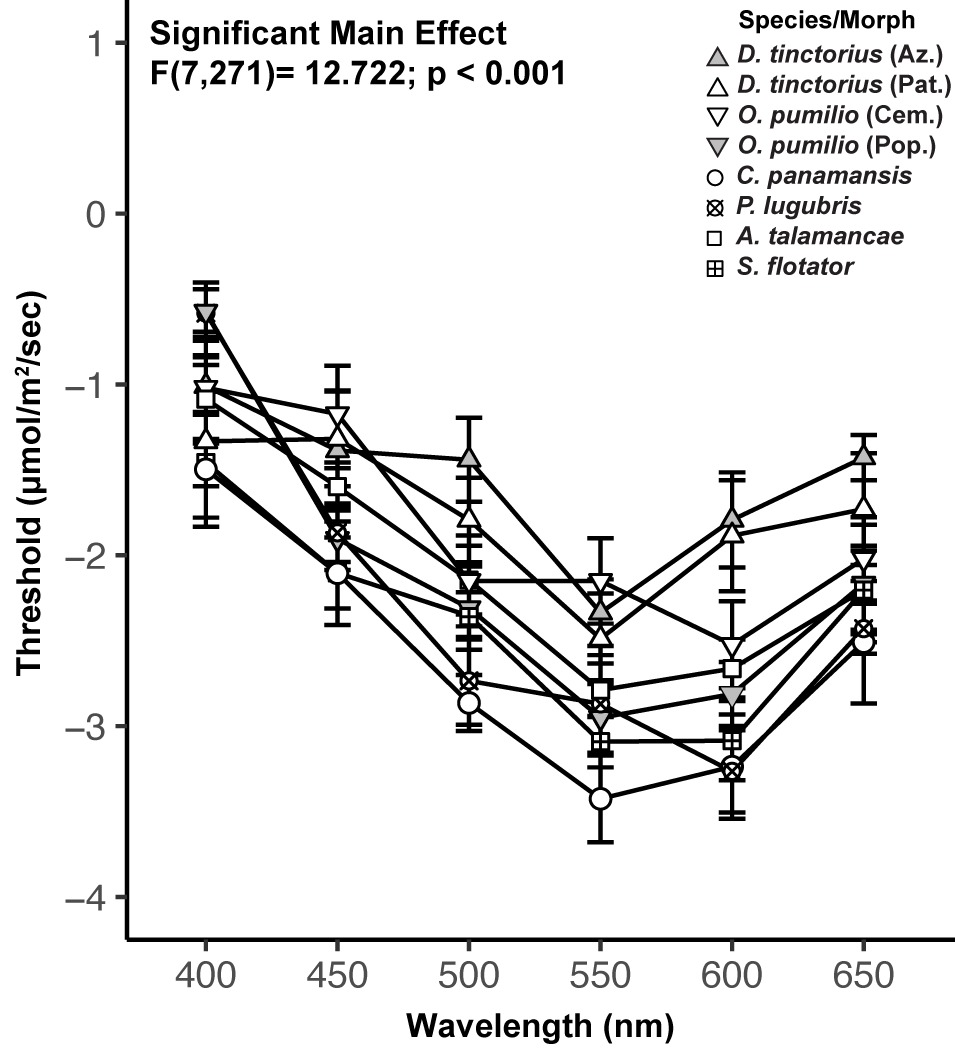

Supplement: S10 Fig — Points indicate the mean threshold response (± S.E.). A statistically significant main effect of species was found. (TIF) [file pone.0312578.s010.tif]

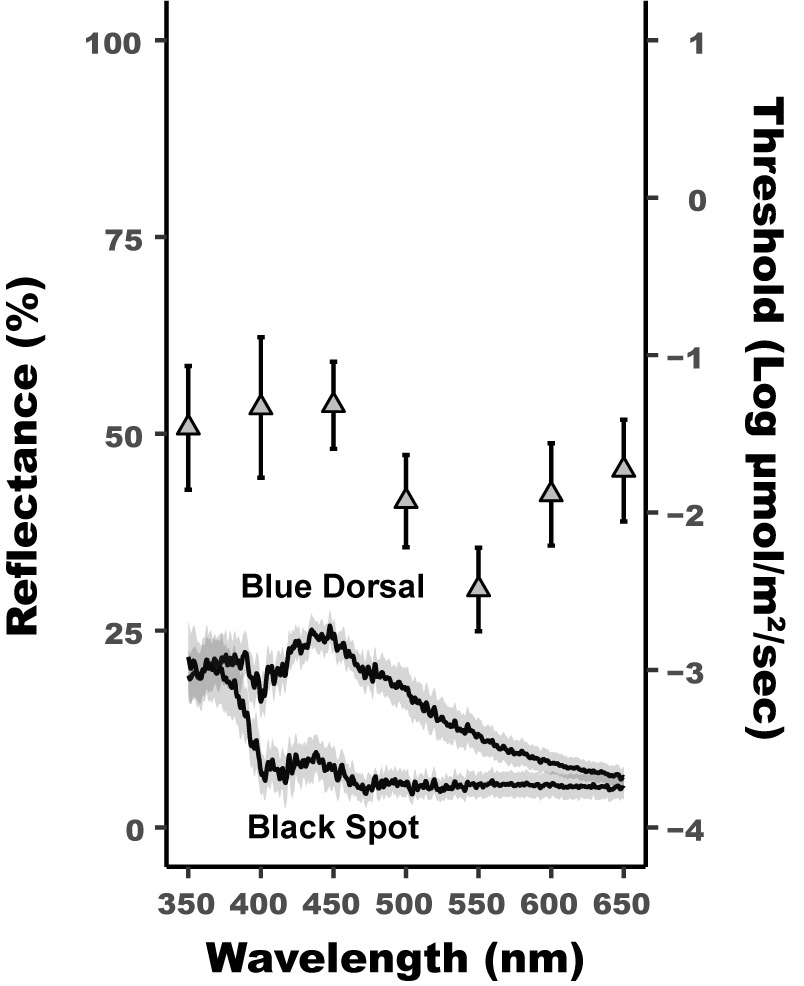

Supplement: S11 Fig — The error bars on the tuning curve points and the shading around the reflectance curve both indicate ± standard error. (TIF) [file pone.0312578.s011.tif]

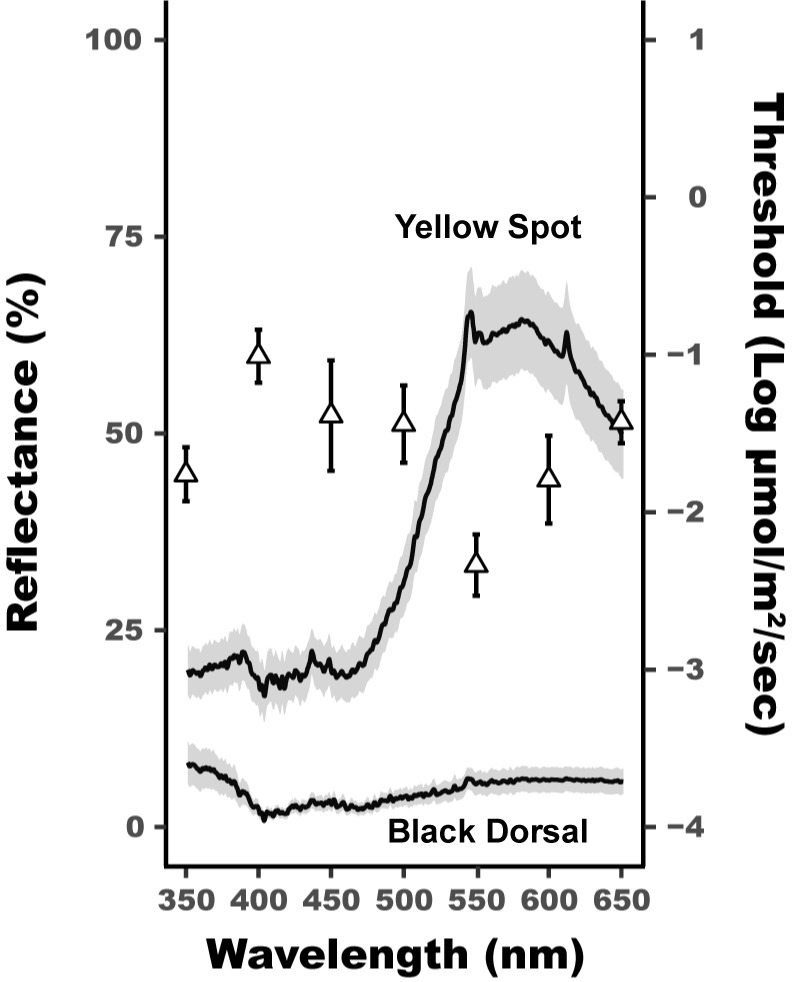

Supplement: S12 Fig — The error bars on the tuning curve points and the shading around the reflectance curve both indicate ± standard error. (TIF) [file pone.0312578.s012.tif]

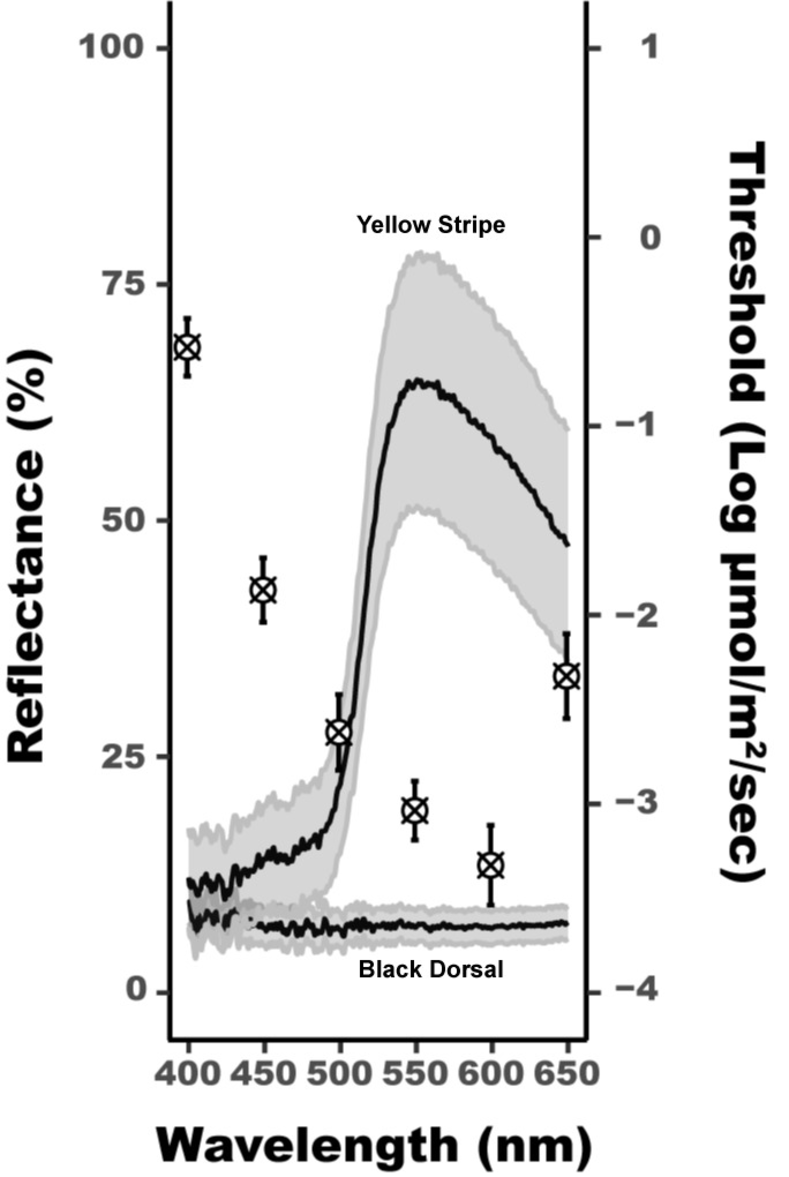

Supplement: S13 Fig — The error bars on the tuning curve points and the shading around the reflectance curve both indicate ± standard error. (TIF) [file pone.0312578.s013.tif]

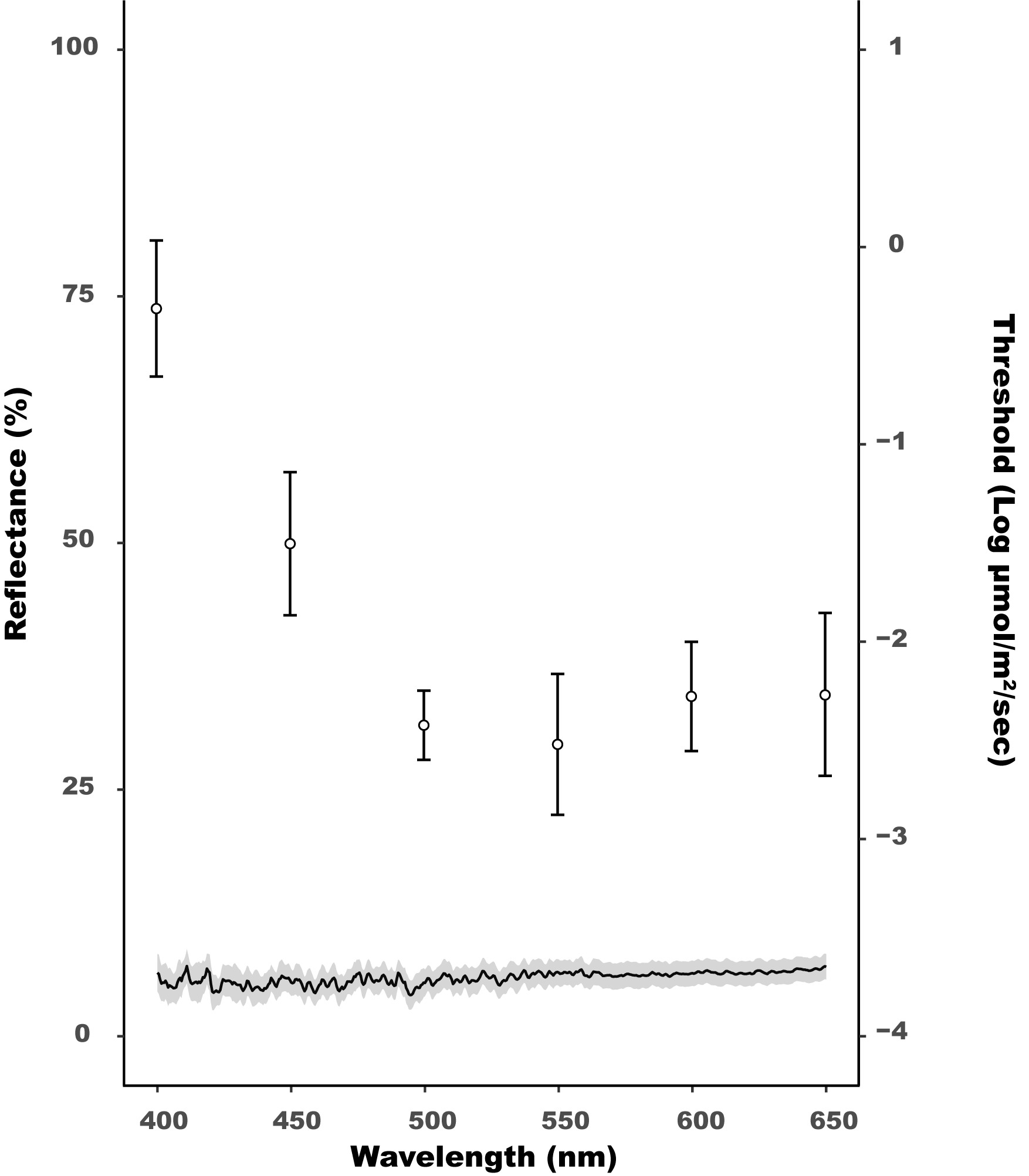

Supplement: S14 Fig — The error bars on the tuning curve points and the shading around the reflectance curve both indicate ± standard error. (TIF) [file pone.0312578.s014.tif]

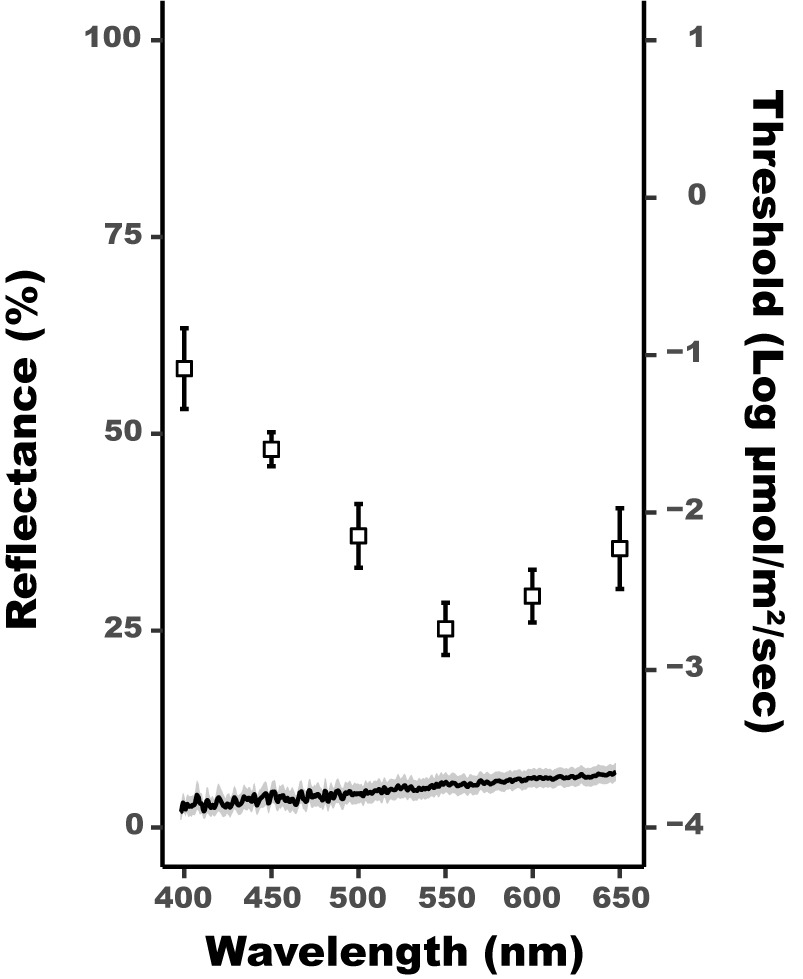

Supplement: S15 Fig — The error bars on the tuning curve points and the shading around the reflectance curve both indicate ± standard error. (TIF) [file pone.0312578.s015.tif]

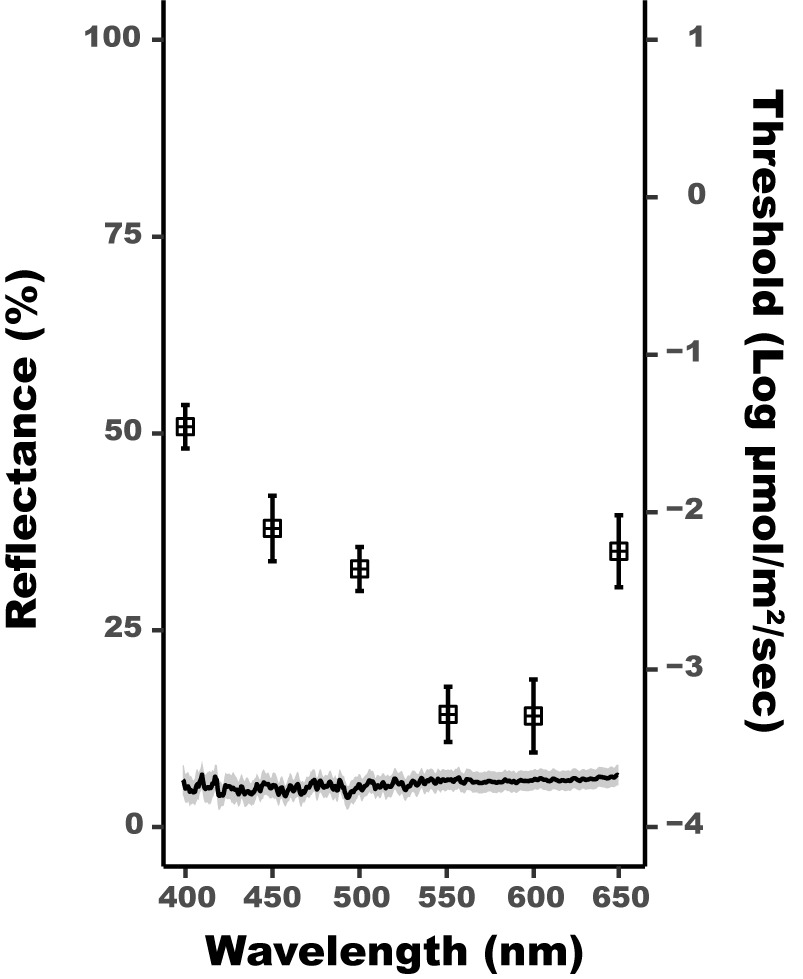

Supplement: S16 Fig — The error bars on the tuning curve points and the shading around the reflectance curve both indicate ± standard error. (TIF) [file pone.0312578.s016.tif]
