# Supplementary material for: The relationship between spectral signals and retinal sensitivity in dendrobatid frogs
Source: PLoS One. 2024 Nov 14;19(11):e0312578. doi: 10.1371/journal.pone.0312578 (PMC11563434; doi:10.1371/journal.pone.0312578)
Supplement: S1 Table — Reflectance curves were gathered for distinct morphological (dorsal, lateral, axillary, and inguinal) regions for each individual specimen. Additional reflectance curves were measured for subregions where there were obvious spectral differences that could be detected by the human eye (i.e. dorsal black and dorsal blue). The data displayed on this table are the mean measurements (± S.E.). (DOCX) [file pone.0312578.s017.docx]

|  | **Region** | **Peak (nm)** | **FWHM (nm)** | **Area Under Curve (% * nm)** | **Average Reflectance (%)** |
| --- | --- | --- | --- | --- | --- |
| **Aposematic** |  |  |  |  |  |
| ***D. tinctorius***  **(Azureus)** |  |  |  |  |  |
|  | **Dorsal**  Black  Blue | 341.25 (±10.3)  425.53 (± 11.6) | 52.6 (± 30.2)  45.7 (± 13.6) | 3531.06 (± 607.1)  5594.86 (± 663.8) | 5.05 (± 1.8)  20.4 (± 2.2) |
|  | **Lateral** | 358.34 (± 4.0) | 69.88 (± 11.2) | 6219.17 (± 446.3) | 10.22 (± 1.8) |
|  | **Axillary** | 480.29 (±16.23) | 73.03 (± 20.4) | 3426.21 (± 868.3) | 13.63 (± 3.4) |
|  | **Inguinal** | 470.4 (± 10.8) | 22.51 (± 4.8) | 1783.12 (± 340.19) | 8.8 (± 2) |
| ***D. tinctorius***  **(Patricia)** |  |  |  |  |  |
|  | **Dorsal**  Black  Yellow | 380.19 (± 11.5)  570.57 (± 7.6) | 34.89 (± 11.5)  188.65 (± 39.4) | 2236.8 (± 637.7)  14327.87 (± 1669.6) | 5.6 (± 1.6)  35.99 (± 3.84) |
|  | **Lateral** | 488.04 (± 24.0) | 38.69 (± 12.3) | 3211.79 (± 510.5) | 11.94 (± 2.8) |
|  | **Axillary** | 486.93 (± 12.25) | 49.42 (± 11.3) | 2716.57 (± 74.55) | 7.73 (± 1.6) |
|  | **Inguinal** | 477.66 (± 13.56) | 56.08 (± 23.9) | 2099.26 (± 355.5) | 5.24 (± 0.88) |
| ***O. pumilio***  **(Cemetery)** |  |  |  |  |  |
|  | **Dorsal**  Black  Red | 534.84 (± 57.4)  641.93 (± 12) | 18.02 (± 0.54)  98.79 (± 2.6) | 959.6 (± 230.8)  7296.99 (± 1236.5) | 0.56 (± 0.54)  7.43 (± 2.6) |
|  | **Lateral** | 645.88 (± 10.21) | 320.25 (± 21.4) | 8634.93 (± 1056.29) | 21.35 (± 3.21) |
|  | **Axillary** | 638.26 (± 19.8) | 247.54 (± 3) | 6639.52 (± 984.5) | 17.7 (± 3) |
|  | **Inguinal** | 647.37 (± 18.94) | 213.05 (±5.08) | 5091.02 (± 1668.9) | 13.64 (± 5.1) |
| ***O. pumilio***  **(Popa)** |  |  |  |  |  |
|  | **Dorsal** | 559.86 (± 7.8) | 140.1 (± 3.1) | 4217.93 (± 906.1) | 16.84 (± 3.1) |
|  | **Lateral** | 585.47 (± 3.9) | 198.5 (± 2.6) | 8494.12 (± 907.8) | 26.53 (± 2.6) |
|  | **Axillary** | 560.4 (± 7.4) | 152.35 (± 4.4) | 5160.52 (± 952.7) | 22.3 (± 4.4) |
|  | **Inguinal** | 587.09 (± 21.8) | 176.35 (± 1.9) | 3910.78 (± 317.1) | 13.14 (± 1.9) |
| **Intermediate** |  |  |  |  |  |
| ***C. panamansis*** |  |  |  |  |  |
|  | **Dorsal** | 373.58 (± 3.6) | 2.4 (± 0.35) | 527.8 (± 83.6) | 4.0 (± 0.82) |
|  | **Lateral** | 535.4 (± 51.29) | 27.22 (± 9.6) | 2084.8 (± 606.4) | 21.6 (± 1.9) |
| (Table Continued) |  |  |  |  |  |
|  | **Axillary** | 546.71 (± 2.3) | 99.24 (± 35.5) | 2102.25 (± 304.1) | 9.81 (± 1.1) |
|  | **Inguinal** | 550.24 (± 4.6) | 146.59 (± 46.5) | 2498.23 (± 462.9) | 12.46 (± 1.8) |
| ***P. lugubris*** |  |  |  |  |  |
|  | **Dorsal**  Black  Yellow | 348.7 (± 5.2)  557.21 (±5.24) | 6.24 (± 1.9)  153.31 (± 10.3) | 2887.92 (± 682.6)  12932.03 (± 3585.7) | 7.1 (± 1.9)  44.09 (± 10.3) |
|  | **Lateral** | 342.59 (± 2.2) | 3.52 (± 2) | 3595.04 (± 659) | 8.41 (± 2) |
|  | **Axillary** | 357.38 (± 2.35) | 2.61 (± 2.8) | 2242.7 (± 675.3) | 7.59 (± 2.8) |
|  | **Inguinal** | 337.5 (± 10.32) | 2.38 (± 1) | 2820.1 (± 178.7) | 9.17 (± 1) |
| **Cryptic** |  |  |  |  |  |
| ***A. talamancae*** |  |  |  |  |  |
|  | **Dorsal** | 493.77 (± 43.8) | 11.01 (± 4.9) | 1768.44 (± 404.3) | 5.93 (± 1.1) |
|  | **Lateral**  Black  White | 447.47 (± 40.1)  513.77 (± 19.1) | 3.67 (± 1.1)  35.37 (± 13.3) | 804.03 (± 183.3)  4489.82 (± 518) | 2.77 (± 0.76)  16.77 (± 2.6) |
|  | **Axillary** | 604.295 (± 14.6) | 61.81 (± 19.2) | 3244.77 (± 427.7) | 9.07 (± 1.4) |
|  | **Inguinal** | 521.38 (± 22.6) | 48.54 (± 19.2) | 4434.69 (± 514) | 11.09 (± 1.28) |
| ***S. flotator*** |  |  |  |  |  |
|  | **Dorsal** | 687.9 (± 2.54) | 16.14 (± 0.97) | 1392.7 (± 287.5) | 3.24 (± 0.97) |
|  | **Lateral**  Black  White | 577.68 (± 52.8)  570.33 (± 29.5) | 13.97 (± 0.74)  73.77 (± 33.8) | 886.67 (± 234.87)  4061.89 (± 412.87) | 1.82 (± 0.74)  10.82 (± 1.5) |
|  | **Axillary** | 620.52 (± 17.7) | 55.97 (± 1.5) | 2543.17 (± 243.8) | 8.13 (± 1.5) |
|  | **Inguinal** | 575.45 (±33.81) | 33.89 (± 0.8) | 2381.62 (± 207.82) | 5.2 (± 0.8) |
