# Supplementary material for: The relationship between spectral signals and retinal sensitivity in dendrobatid frogs
Source: PLoS One. 2024 Nov 14;19(11):e0312578. doi: 10.1371/journal.pone.0312578 (PMC11563434; doi:10.1371/journal.pone.0312578)
Supplement: S2 Table — D. tinctorius (D. t.), O. pumilio (O. p.), C. panamansis (C. p.), P. lugubris (P. l.), A. talamancae (A. t.), and S. flotator (S. f.). (DOCX) [file pone.0312578.s018.docx]

| **Species** | ***D. t.*** | ***O. p.*** | ***C. p.*** | ***P. l.*** | ***A. t.*** | ***S. f.*** |
| --- | --- | --- | --- | --- | --- | --- |
| ***D. t.*** | - | *  p = 0.04 | ***  p < 0.001 | **  p < 0.01 | *  p = 0.02 | ***  p < 0.001 |
| ***O. p.*** |  | - | ***  p < 0.001 | N.S. | N.S. | **  p < 0.01 |
| ***C. p.*** |  |  | - | N.S. | *  p = 0.02 | N.S. |
| ***P. l.*** |  |  |  | - | N.S. | N.S. |
| ***A. t.*** |  |  |  |  | - | N.S. |

N.S. (Not Significant); * (p < 0.05); ** (p < 0.01); *** (p < 0.001)
