# Supplementary material for: The relationship between spectral signals and retinal sensitivity in dendrobatid frogs
Source: PLoS One. 2024 Nov 14;19(11):e0312578. doi: 10.1371/journal.pone.0312578 (PMC11563434; doi:10.1371/journal.pone.0312578)
Supplement: S4 Table — D. tinctorius (D. t. Az. And Pat.), O. pumilio (O. p. Cem. and Pop.), C. panamansis (C. p.), P. lugubris (P. l.), A. talamancae (A. t.), and S. flotator (S. f.). (DOCX) [file pone.0312578.s020.docx]

| **Species** | **F statistic** | **p value** |
| --- | --- | --- |
| ***D. t.* Az.** | F(1,20)= 1.22 | 0.28 |
| ***D.t.* Pat** | F(1,30)= 0.023 | 0.88 |
| ***O.p.* Cem** | F(1,26)= 1.496 | 0.23 |
| ***O. p.* Pop.** | F(1,51)= 0.296 | 0.58 |
| ***C. p.*** | F(1, 27) = 2.612 | 0.12 |
| ***P. l.*** | F(1,23)= 0.382 | 0.54 |
| ***A. t.*** | F(1, 33)= 1.303 | 0.26 |
| ***S. f.*** | F(1, 53) = 0.003 | 0.95 |
